# Enterocyte-Associated Microbiome of the Hadza Hunter-Gatherers

**DOI:** 10.3389/fmicb.2016.00865

**Published:** 2016-06-06

**Authors:** Silvia Turroni, Simone Rampelli, Manuela Centanni, Stephanie L. Schnorr, Clarissa Consolandi, Marco Severgnini, Clelia Peano, Matteo Soverini, Mirella Falconi, Alyssa N. Crittenden, Amanda G. Henry, Patrizia Brigidi, Marco Candela

**Affiliations:** ^1^Microbial Ecology of Health Unit, Department of Pharmacy and Biotechnology, University of BolognaBologna, Italy; ^2^Max Planck Research Group on Plant Foods in Hominin Dietary Ecology, Max Planck Institute for Evolutionary AnthropologyLeipzig, Germany; ^3^Institute of Biomedical Technologies, Italian National Research CouncilSegrate, Italy; ^4^Department of Biomedical and Neuromotor Sciences, University of BolognaBologna, Italy; ^5^Metabolism, Anthropometry, and Nutrition Laboratory, Department of Anthropology, University of Nevada, Las VegasNV, USA

**Keywords:** gut microbiota, enterocyte-associated microbiome, microbiota-host interactions, Hadza hunter-gatherers, human evolution

## Abstract

By means of a recently developed non-invasive *ex vivo* minimal model based on the interaction of the human enterocyte-like HT29 cell line and fecal slurries, we explored the enterocyte-associated microbiome of 21 Hadza hunter-gatherers and nine urban living Italians. Though reductionist, this model allows inferring the microbiota structural and functional arrangement as it interacts with enterocytes. Microbial suspensions obtained from Hadza or Italian stools were first evaluated for structural integrity by high resolution-scanning electron microscopy and co-incubated with HT29 cell monolayers. The enterocyte adherent microbiota fraction was then characterized by 16S rRNA gene sequencing and predictive functional profiling using PICRUSt. Compared to Italians, the Hadza enterocyte-associated microbiome was characterized by a greater amount of adhesive microorganisms with pathogenic potential, such as Proteobacteria, *Erysipelotrichaceae*, *Enterococcus*, *Clostridium* and *Sarcina*. These compositional characteristics were reflected in a functional enrichment in membrane transport, signal transduction, signaling molecules and interaction. Our results depict a new interesting mutualistic configuration of the enterocyte-associated microbiome in Hadza, stressing the importance of microbe-host interaction at the mucosal surface along the course of human evolution.

## Introduction

We recently explored the fecal microbial community of Hadza hunter-gatherers from Tanzania (Schnorr et al., 2014; Rampelli et al., 2015), one of the last remaining hunting and gathering communities in the world (Blurton Jones et al., 1992). According to these previous findings, the structural and functional configuration of the Hadza gut microbiota is well aligned with the dietary and environmental factors characteristic of their foraging lifestyle, supporting the importance of microbiota as an evolutionary legacy that provides specific adaptive versatility to disparate human subsistence and environmental occupation. Along with a high ecosystem diversity and the great fibrolytic potential, the Hadza gut microbiota was enriched in microorganisms commonly considered to be opportunistic pathogens – e.g., *Treponema*, Proteobacteria, and Spirochaetes – while lacking health-promoting bifidobacteria (Schnorr et al., 2014). From a functional point of view, the Hadza gut microbiota was adapted for broad-spectrum carbohydrate metabolism, and equipped for branched-chain amino acid degradation and aromatic amino acid biosynthesis (Rampelli et al., 2015). This structural and functional gut microbiota layout represents a new mutualistic microbiota-host equilibrium that possibly co-evolved to cope with the Hadza foraging subsistence strategy.

In an attempt to provide some glimpse into the Hadza-microbiota cross-talk at the intestinal surface, here we investigated the compositional and functional potential of the enterocyte adherent fraction of fecal-derived gut microbiota from 21 Hadza hunter-gatherers compared to nine urban living Italian adults. Microorganisms that directly interact with the enterocyte surface have a primary role in the microbiota-host cross-talk (Sansonetti, 2011; Wells et al., 2011), and structural moieties and metabolites derived from these microorganisms can mold functions of the host epithelium and other cell types in the mucosa (Kabat et al., 2014). Indeed, microbial products have been reported to modulate the enterocyte barrier function and mucus structure (Hooper and Macpherson, 2010). Moreover, microbial components of the adherent microbiome have been shown to control defensin secretion, wound healing (Gallo and Hooper, 2012) and, more importantly, the functioning of the adaptive immune system (Maynard et al., 2012).

To infer the enterocyte-associated microbiome of Hadza hunter-gatherers, we employed a recently developed non-invasive *ex vivo* minimal model based on the human enterocyte-like HT29 cell line (Centanni et al., 2013). Despite some limitations, including their heterogeneity and the generally low proportion of mucus-secreting cells, HT29 cells were selected because they are considered among the most relevant models to reproduce *in vitro* the intestinal mucosa (Rousset, 1986; Neutra and Louvard, 1989). Since the fecal microbiota consists of both luminal and mucosal components (Eckburg et al., 2005; Walter and Ley, 2011), our model involved the co-incubation of HT29 cell monolayers with microbial suspensions obtained from Hadza or Italian stools, followed by the characterization of the enterocyte adherent microbiota fraction by 16S rRNA gene sequencing and predictive functional profiling using PICRUSt (Langille et al., 2013). Although we used a reductionist approach that might miss some mucosa-associated microbial members, our data allowed us to uncover a peculiar configuration of the enterocyte-associated microbiome from Hadza, suggesting the existence of community specific adaptive factors contributing to the microbial arrangement at the interface of the intestinal epithelium.

## Materials and Methods

### Subjects and Sample Collection

Stool samples from 21 Hadza hunter-gatherers of Tanzania and nine urban living Italian adults from the study cohort of Schnorr et al. (2014) were used in the present study. Hadza were from the Dedauko and Sengele camps in northwestern Tanzania (age: 13–70 years; mean, 33). Their fecal samples were frozen immediately after collection at -20°C (Engel MHD13F-DM field freezer) and shipped on dry ice to Italy. Italian adults (age: 25–38 years; mean, 31) were from the greater Bologna metropolitan area. They were asked to collect one fecal sample, store it at -20°C and bring it to the research laboratory within 24–48 h. All fecal samples were stored at -80°C until use. Informed consent was obtained from all the subjects enrolled. Since Hadza are non-literate, verbal consent was obtained by those who agreed to participate, and this was documented by a separate witness. In the case of young Hadza, we obtained verbal assent from the youths and verbal consent from the parents, which was again documented by a separate witness. The study was conducted according to the principles expressed in the Declaration of Helsinki. All experimental protocols were approved by the University of Leipzig Ethik-kommission review board, reference number 164-12-21052012. Permission for this work was granted from the Tanzanian Commission for Science and Technology (COSTECH), permit number 2012-315-NA-2000-80, and the National Institute for Medical Research. Methods were carried out in accordance with the approved guidelines.

### Fecal Slurry-HT29 Cell Interaction Assay

Fecal slurries were rapidly prepared by diluting stools 1:2 in ice-cold Dulbecco’s modified Eagle’s medium (DMEM; Sigma–Aldrich, St. Louis, MO, USA) followed by Stomacher blending, as in Centanni et al. (2013). The number of viable bacterial cells in fecal slurries was determined by plate counting on Wilkins-Chalgren Agar (WCA; Oxoid Limited, Hampshire, UK) and Nutrient Agar (NA; Difco, BD, Franklin Lakes, NJ, USA) for total anaerobes and facultative anaerobes, respectively. Samples were appropriately diluted in pre-reduced Ringer’s solution (Oxoid Limited). WCA plates were incubated in an anaerobic chamber (Concept 400, Ruskinn Technology, South Wales, UK) at 37°C under an atmosphere of 85% N_2_, 10% CO_2_, and 5% H_2_ for up to 7 days. NA plates were incubated aerobically for the same time at 37°C. Microbial structural integrity was evaluated by high resolution-scanning electron microscopy (HR-SEM) as previously reported (Teti et al., 2012). In brief, fecal slurries were fixed with a solution of 2.5% glutaraldehyde in 0.1 M phosphate buffer (Sigma–Aldrich) for 2 h at 4°C and subsequently post-fixed with 1% OsO_4_ in 0.1 M phosphate buffer (Società Italiana Chimici, Rome, Italy) for 1 h at RT. After several washings in 0.15 M phosphate buffer, samples were dehydrated in an ascending alcohol series and critical point dried (CPD 030, Balzers, Leica Microsystems GmbH, Wetzlar, Germany). Then, samples were observed under HR-SEM (JSM 890, Jeol Company, Tokio, Japan) with 7 kV accelerating voltage and 1 × 10^-11^ mA.

The human colonic epithelial HT29 cells were routinely grown in DMEM with 4.5 g/l glucose supplemented with 10% heat-inactivated fetal bovine serum (FBS; Sigma–Aldrich), 1% L-glutamine (Sigma–Aldrich) and 1% penicillin-streptomycin (Sigma–Aldrich), in a humidified 5% CO_2_ atmosphere at 37°C, as reported by O’Hara et al. (2006).

Fecal slurry-HT29 cell interaction assays were performed as previously described (Centanni et al., 2013). Briefly, 2.5 × 10^5^ HT29 cells were seeded per well in 24-well tissue culture plates (BD Falcon, Becton Dickinson, Heidelberg, Germany), and allowed to grow to confluent monolayers. Twenty-four hours before the interaction, the cell medium was replaced with DMEM, 25 mM HEPES, 1 g/l glucose (Gibco BRL, Life Technologies, Carlsbad, CA, USA), and 1% FBS. On the assay day, 1 ml of ice-cold DMEM-diluted fecal slurry, containing 10^10^ viable bacterial cells, was rapidly added to the HT29 cell monolayers and incubated at 37°C and 5% CO_2_ for 1 h. Wells were washed thrice with phosphate buffered saline (PBS), and cells and adherent microorganisms were detached after a 10-min incubation at 37°C with 0.05% trypsin/0.02% EDTA (Sigma–Aldrich), followed by a further PBS rinsing. Samples were kept at -20°C until DNA extraction.

To exclude that there was bacterial growth during the fecal slurry-HT29 cell interaction, fecal slurries were incubated under the same conditions of the interaction assay but in the absence of HT29 cell monolayers, and analyzed for facultative and obligate anaerobic bacterial counts as described above.

### 16S rRNA Gene Sequencing and Processing

Total microbial DNA was extracted using DNeasy Blood & Tissue Kit (QIAGEN, Hilden, Germany) with a modified protocol incorporating a FastPrep (MP Biomedicals, Irvine, CA, USA) bead-beating step (Centanni et al., 2013). Genomic DNA concentration and quality were evaluated using NanoDrop ND-1000 spectrophotometer (NanoDrop Technologies, Wilmington, DE, USA) and 2200 TapeStation instrument (Agilent Technologies, Santa Clara, CA, USA).

For each sample, the V3–V4 region of the 16S rRNA gene was PCR amplified, and the resulting single amplicons of approximately 460 bp were cleaned up and sequenced on Illumina MiSeq platform using a 2 × 300 bp paired end protocol, according to the manufacturer’s instructions (Illumina, San Diego, CA, USA). In detail, PCR reactions were carried out in 25 μl volumes containing 12.5 ng of microbial DNA, 2x KAPA HiFi HotStart ReadyMix (KAPA Biosystems, Resnova, Rome, Italy), and 200 nM of S-D-Bact-0341-b-S-17/S-D-Bact-0785-a-A-21 primers (Klindworth et al., 2013) carrying Illumina overhang adapter sequences. Reaction conditions were as follows: initial denaturation at 98°C for 3 min, followed by 25 cycles of denaturation at 95°C for 30 s, annealing at 55°C for 30 s, and extension at 72°C for 30 s, with a final extension step at 72°C for 5 min. Amplicons were purified with a magnetic bead-based clean-up system (Agencourt AMPure XP; Beckman Coulter, Brea, CA, USA). Indexed libraries were prepared by limited-cycle PCR using Nextera technology and further cleaned up with Agencourt magnetic beads. The final libraries were pooled at equimolar concentrations, denatured and diluted to 6 pM before loading onto the MiSeq flow cell.

Raw sequences were processed using a pipeline combining PANDAseq (Masella et al., 2012) and QIIME (Caporaso et al., 2010). After length and quality filtering with default parameters, reads were binned into OTUs at a 0.97 similarity threshold using UCLUST (Edgar, 2010). Taxonomy was assigned using the RDP classifier against Greengenes database (May 2011 release). To filter out PCR errors and chimeras, all singleton OTUs were discarded. Alpha rarefaction was performed using the Faith’s phylogenetic diversity, Chao1, observed species, and Shannon index metrics. Beta diversity was estimated by computing weighted and unweighted UniFrac distances, which were used as input for principal coordinates analysis (PCoA). Age-discriminatory bacterial taxa were determined using the Random Forests machine learning algorithm (Breiman, 2001). Briefly, the relative abundances of bacterial genera were regressed against subject age, and the significant age-discriminatory taxa were identified by comparing fitted to null models, where ages were randomly permuted 9999 times with respect to the microbial profiles.

Metagenome imputation of Greengenes-picked OTUs was performed using PICRUSt (Langille et al., 2013) with default settings. The KEGG Orthology database (Kanehisa et al., 2012) was used for functional annotation.

All statistical analyses were performed in R 3.1.1. *P-*values were corrected for multiple comparisons using the Benjamini–Hochberg method when appropriate. A corrected *P* < 0.05 was considered as statistically significant.

Sequencing reads were deposited in MG-RAST (http://metagenomics.anl.gov/linkin.cgi?project=12183).

## Results

### Structure and Diversity of the Enterocyte-Associated Microbiota in Hadza and Italians

The enterocyte adherent fraction of the gut microbiota from 21 Hadza hunter-gatherers compared to nine urban living Italian adults, was investigated using a recently developed non-invasive *ex vivo* reductionist approach, based on the interaction of fecal slurries and HT29 cell monolayers (Centanni et al., 2013). First, the structural integrity of microbial cells in fecal slurries was evaluated by HR-SEM. As shown in the micrographs of **Supplementary Figure S1**, fecal bacteria were intact without any sign of damage to the cell envelope. Average viable cell counts of fecal slurries on Wilkins-Chalgren Agar targeting total anaerobes and Nutrient Agar targeting total facultative anaerobes, were 10.7 ± 0.5 log CFU/g and 7.9 ± 0.8 log CFU/g feces, respectively. For each subject, 10^10^ total viable bacterial cells in ice-cold DMEM were added to HT29 cell monolayers and incubated as in standard bacterial adhesion assays (Candela et al., 2008). To rule out a potential bias due to preferential growth of facultative over obligate anaerobes during the assay conditions, viable cell counts were repeated after incubation of fecal slurries under the same conditions of the interaction assay but in the absence of HT29 cells. As expected, no difference was observed either in the anaerobic or oxygen-tolerant bacterial fraction (data not shown). HT29 cell-associated bacterial cells were then characterized by 16S rRNA gene sequencing and compared with the respective fecal microbial communities (Schnorr et al., 2014).

A total of 952,376 high-quality sequence reads (mean per subject, 31,746; range, 6,415–52,217; average length, 448 bp) were obtained and analyzed. Reads were clustered into 15,671 non-singleton OTUs at 97% similarity. According to our data, the HT29 cell-associated microbiota fractions did not simply reflect the slurry microbiota structure, rather they were significantly different from the fecal counterpart (*P* = 0.0001, permutation test with pseudo-F ratios) (**Supplementary Figure S2**), proving a rearrangement of the microbial communities at the enterocyte surface, for both the Hadza and Italians, during the interaction assay in our *ex vivo* minimal model. The enterocyte-associated microbiota fractions of the two populations distinctly segregated from each other (*P* = 0.004), suggesting the existence of different microbial configurations at the epithelial interface, possibly resulting in different cross-talk interactions with the host. The enterocyte adherent microbiota of Hadza clustered separately from the Italian one, also according to both weighted and unweighted UniFrac metrics (*P* = 0.0001) (**Supplementary Figure S3**). In particular, a larger portion of variance in the first two principal coordinate axes is accounted for by weighted UniFrac (56.3% vs. unweighted UniFrac, 11.3%), indicating that abundance information is particularly relevant in differentiating the microbial community structures. Compared to Italians, Hadza exhibit a higher degree of inter-individual diversity in the microbiota adhering to the intestinal surface (mean weighted UniFrac distance ± SD, Hadza, 0.334 ± 0.107 vs. Italians, 0.238 ± 0.059; *P* < 0.001, Wilcoxon-Mann-Whitney rank sum test). It cannot be excluded that the age disparity between Hadza (age range, 13–70) and Italians (age range, 25–38) contributes, at least in part, to the observed differences in the microbiota diversity. However, according to a regression analysis of the genus relative abundances against subject age using Random Forests (Breiman, 2001), only 5, very subdominant bacterial taxa were found to be age-discriminatory (*Balneimonas*, *Bavariicoccus*, unclassified *Marinilabiaceae*, and unclassified members belonging to the Actinobacteria and Clostridia classes; cumulative relative abundance per subject, mean ± SD, 0.08 ± 0.06; *P* ≤ 0.02, 9999 permutations), suggesting a minor effect of age on the microbial structure across our study cohort.

**FIGURE 1:**
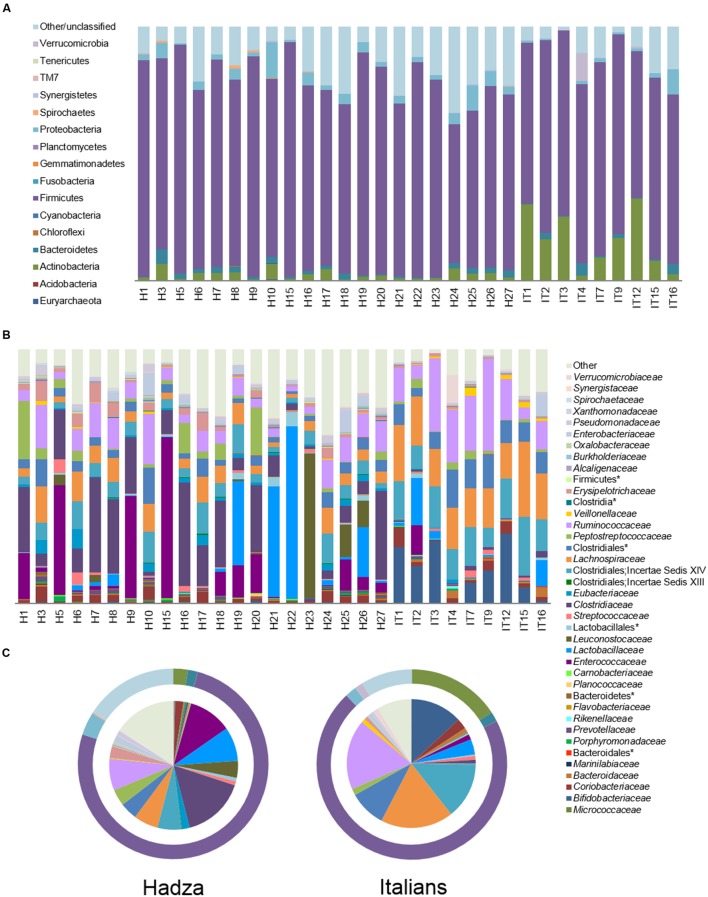
**Enterocyte-associated microbiota in Hadza and Italians.** Relative abundance of enterocyte adherent bacterial phyla **(A)** or families **(B)** in 21 Hadza (H) and nine Italians (I). **(C)** Pie charts summarizing family level taxa. Outer rings depict phylum level distribution. Only families with a mean relative abundance ≥0.1% in at least one of the two populations are shown. ^∗^denotes unclassified OTU reported at higher taxonomic level.

Analysis of the relative taxon abundance confirmed the divergence between the structure of the fecal microbiota and that of the microbial communities adhering to the enterocytes, and identified many notable differences between the Hadza and Italian HT29 cell-associated microbiota at phylum and family level (**Figure 1** and **Supplementary Figure S2**). Although Firmicutes largely dominate the enterocyte adherent microbiota in both populations (Hadza, 75.8% vs. Italians, 70.5%), Hadza are characterized by a considerably lower abundance of Actinobacteria (2.4% vs. Italians, 15.8%; *P* = 0.0004, Wilcoxon-Mann-Whitney rank sum test) and a higher abundance of Proteobacteria (3.9% vs. Italians, 2.0%; *P* = 0.004). Notably, 15.5% of phylum level OTUs in the Hadza enterocyte-associated microbiota remain unclassified, compared to 8.6% for Italians (*P* = 0.02). The most represented families are *Clostridiaceae* (16.0%) and *Enterococcaceae* (10.9%) for Hadza (*P* ≤ 0.0004), and *Lachnospiraceae* (18.3%), *Ruminococcaceae* (17.6%), Clostridiales Incertae Sedis XIV (14.0%) and *Bifidobacteriaceae* (13.0%) for Italians (*P* ≤ 0.007). The enterocyte adherent microbiota fractions of Hadza and Italians also differed within subdominant families (<10% relative abundance). Specifically, Hadza were enriched in *Erysipelotrichaceae* (2.9% vs. Italians, 0.8%), *Eubacteriaceae* (1.9% vs. Italians, 0.3%), *Enterobacteriaceae* (1.7% vs. Italians, 1.0%), *Pseudomonadaceae* (1.2% vs. Italians, 0.6%) and *Prevotellaceae* (0.6% vs. Italians, 0.2%), and correspondingly depleted in *Bacteroidaceae* (0.2% vs. Italians, 1.1%), *Veillonellaceae* (0.3% vs. Italians, 1.0%) and unclassified Clostridiales (4.3% vs. Italians, 9.2%; *P* ≤ 0.04).

**FIGURE 2:**
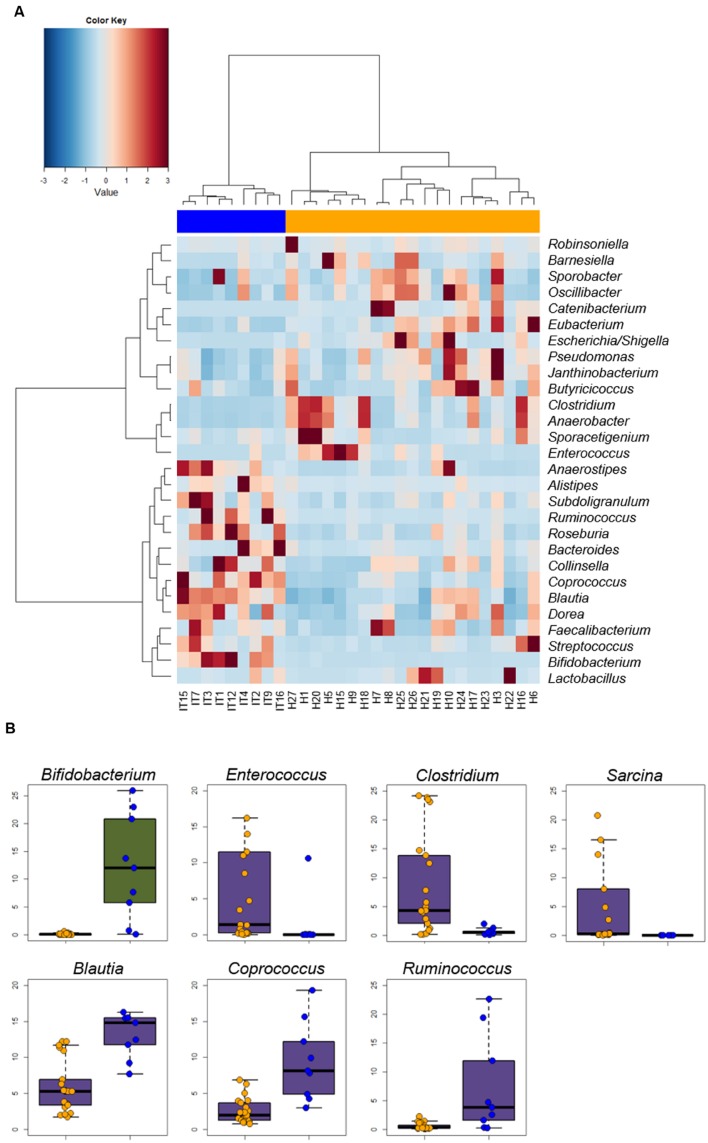
**Genus-level comparison between Hadza and Italian microbiota adhering to the intestinal epithelial surface.** **(A)** Heat map of the core microbiota, including 28 genera that occurred in all subjects with a mean relative abundance of at least 0.1% in either Hadza or Italians. Hierarchical clustering was performed using a Spearman’s correlation-based dissimilarity metrics and Ward’s agglomeration method. *P* = 7E-8, Fisher’s exact test. **(B)** Strip charts and box plots showing the relative abundance of genera significantly differing between Hadza and Italians (*P* < 0.05, Wilcoxon-Mann-Whitney rank sum test). Only genera contributing ≥5% in at least one population are plotted. Box color indicates phylum membership as in **Figure 1A**. Hadza, orange; Italians, blue. See also **Supplementary Table S1**.

At the genus level, we could identify a core microbiota residing on the intestinal epithelial surface, comprising 28 genera that occurred in all subjects and whose mean relative abundance was at least 0.1% in Hadza or Italians (**Figure 2A**). These core genera account for 55.4% and 67.8% of the total sequences in the enterocyte-associated microbiota of Hadza and Italians, respectively. The majority of these genera belong to the Firmicutes phylum, and mainly the Clostridiales order. Although common to all samples, the abundance of these core genera varied greatly between the two populations (**Figure 2B**, **Supplementary Table S1**). The enterocyte-associated microbiota of Hadza shows a remarkably higher abundance of *Catenibacterium*, *Escherichia*/*Shigella*, *Clostridium*, *Anaerobacter*, *Enterococcus*, *Eubacterium*, *Barnesiella*, *Oscillibacter*, *Pseudomonas*, and *Janthinobacterium*, which were detected in the Italian epithelial microbiota at only 0.02–1.2% level of abundance (*P* ≤ 0.04). On the other hand, Italians were comparatively enriched in a number of *Clostridium* cluster IV and XIVa components, as *Blautia*, *Coprococcus*, *Ruminococcus*, *Dorea*, *Roseburia*, *Subdoligranulum* and *Anaerostipes*, as well as in *Alistipes* and particularly *Bifidobacterium* (*P* ≤ 0.04). In addition to these core components, the genera *Sarcina*, *Slackia*, *Allobaculum* and *Bulleidia* are all extremely rare if not absent in Italians, but were found to be overrepresented within the microbial communities at the enterocyte surface of all the Hadza sampled. In particular, *Sarcina* stands out at a 6.4% relative abundance (Italians, 0.01%; *P* = 0.00001) (**Figure 2B**, **Supplementary Table S1**). Interestingly, *Sarcina* abundance was found to significantly differ by sex in the Hadza, with an average of 15.1% in the enterocyte-associated microbiota of women versus only 2.1% in men (*P* = 0.04). Compared to men, the Hadza woman microbiota on the intestinal epithelial surface was also remarkably enriched in the subdominant genera *Treponema* and *Oribacterium*, and depleted in *Eubacterium*, *Slackia* and *Pediococcus* (*P* ≤ 0.04) (**Supplementary Table S2**). Conversely, no evidence of a sex-related divergence in the enterocyte adherent microbiota structure of Italians was found (**Supplementary Figure S4**).

### Predicted Functional Potential of Hadza and Italian Microbial Communities at the Intestinal Epithelial Interface

To explore the functional contribution of the Hadza and Italian microbial communities adhering to the intestinal epithelial surface, we employed a computational approach, PICRUSt (Langille et al., 2013), for inferring metagenomics functions from phylogenetic profiles.

Similar to the phylogenetic counterpart, PICRUSt-imputed metagenomes of the enterocyte-associated microbiota of Hadza and Italians do not differ for functional diversity but cluster separately in the PCoA plot, in particular along the first component for Jaccard distances (*P* = 0.009, Wilcoxon-Mann-Whitney rank sum test), and the second component for Euclidean and Bray–Curtis distances (*P* = 0.02) (**Supplementary Figure S5**). Consistent with this, the Euclidean hierarchical clustering shows a functional divergence between Hadza and Italian epithelial microbiota (*P* < 0.000001, Fisher’s exact test) (**Supplementary Figure S6**).

Looking at the top 100 most represented KEGG orthologs (KOs) in the enterocyte adherent microbial communities of Hadza, we saw that the majority (37 KOs) were transport systems, followed by several enzymes acting on different substrates in metabolic pathways, and proteins involved in genetic information processing (**Supplementary Table S3**). Specifically, we observed transporters for sugars, amino acids or short peptides, mineral and organic ions, and metals, especially nickel. Among pathway modules, we recovered primarily functional units from nucleotide and amino acid metabolism, followed by carbohydrate and lipid, and energy. We also identified many transcription factors, replication and repair proteins, chaperones and folding catalysts. We additionally found signal transduction components of the two-component regulatory system, along with a percentage of uncharacterized proteins.

**FIGURE 3:**
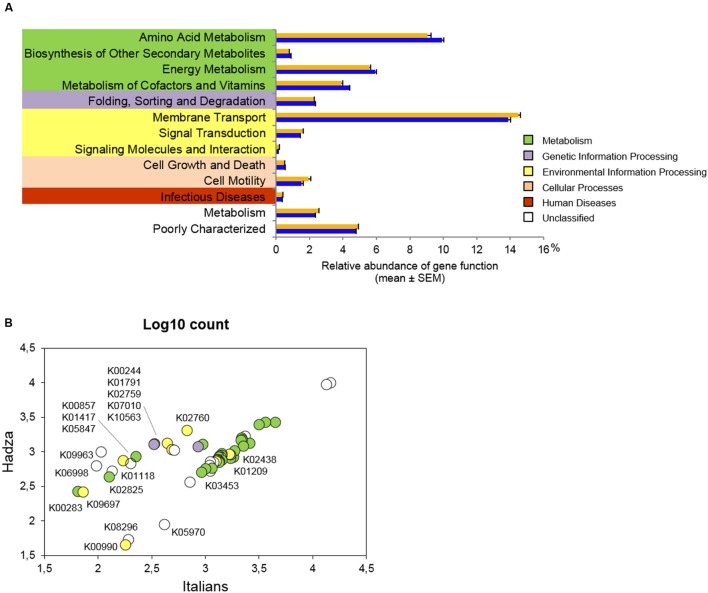
**Predicted functional potential of the enterocyte-associated microbiome in Hadza and Italians.** **(A)** KEGG pathways significantly differentially abundant between Hadza and Italians. Only pathways with a mean proportion ≥0.1% in at least one population are plotted. Hadza, orange; Italians, blue. **(B)** Individual KEGG orthologs (KOs) that significantly differ between Hadza and Italians. Log_10_ counts are colored by pathway membership as in **(A)**, and assigned to the KO identifier when the fold change between Hadza and Italians was at least 2. *P* < 0.05, Wilcoxon-Mann-Whitney rank sum test. See also **Supplementary Tables S4** and **S5**.

At the second highest level of functional categories, 13 KEGG pathways significantly differ in abundance between Hadza and Italians (**Figure 3A**, **Supplementary Table S4**). Metabolic pathways, such as amino acid, energy, cofactor and vitamin metabolism, as well as biosynthesis of other secondary metabolites, were found to be overrepresented in the enterocyte-associated microbiome of Italians (*P* ≤ 0.002, Wilcoxon-Mann-Whitney rank sum test). Conversely, predicted functionality in the Hadza microbial communities on the intestinal epithelial surface is represented by cell motility as well as pathways linked to environmental information processing, including membrane transport, signal transduction, signaling molecules and interaction (*P* ≤ 0.03). Also pathways related to infectious diseases and unclassified functions are more highly represented in the enterocyte adherent microbiome of Hadza (*P* ≤ 0.002).

At the level of individual genes, we grouped the KO identifiers that were imputed with a count ≥1 in all the Hadza and Italians sampled, thus defining a core metagenome at the intestinal epithelial surface. Clustering analysis of the core metagenome, comprising 2927 of the 5827 microbial genes inferred in the whole dataset, failed to show a clear separation between Hadza and Italian samples, reflecting an overall similarity in core functional abundances among the community memberships (**Supplementary Figure S7**). Despite this, 56 core functions were found to be significantly different in abundance between the two populations, and 17 of these (30%) were overrepresented in Hadza samples (**Figure 3B**, **Supplementary Table S5**). After sorting the Hadza overabundant KOs in descending order of fold change, we first found a hypothetical protein-encoding gene enriched by a factor of 9 in the microbial communities of Hadza compared to Italians (*P* = 0.008, Wilcoxon-Mann-Whitney rank sum test), followed by a KO gene encoding for PhzF, an enzyme essential for phenazine biosynthesis, that is seven times more abundant in the Hadza enterocyte adherent microbiome (*P* = 0.04). Furthermore, Hadza samples are predicted to be enriched 2–4 fold in genes encoding structural complexes for the membrane transport of organic osmolytes, sodium or cellobiose, as well as metabolic enzymes, and proteins involved in genetic information processing (*P* ≤ 0.04).

On the other hand, the enterocyte-associated metagenome of Italians shows an overall enrichment of genes involved in carbohydrate transport and metabolism and amino acid metabolism (**Figure 3B**, **Supplementary Table S5**). Among these, we note a peculiar overrepresentation of a number of glycosidases acting on different polymeric backbones (*P* ≤ 0.02). Moreover, as anticipated from the comparison of level 2 KEGG categories, we found an increased abundance for synthases and transferases directly involved in the biosynthetic pathways of amino acids (*P* ≤ 0.01). In this regard, we specifically retrieved the *trp*A to *trpF* structural genes of the tryptophan operon (*P* ≤ 0.003).

## Discussion

According to our findings, the Hadza microbial communities at the intestinal epithelial interface showed a peculiar structural configuration that was considerably enriched in what we generally consider to be potentially pathogenic microorganisms, mirroring in part what has already been documented in their fecal microbiota (Schnorr et al., 2014). Besides the increased abundance of Proteobacteria, the Hadza enterocyte-associated microbial communities were indeed characterized by an overrepresentation of other so-termed pathobionts, including especially members of *Erysipelotrichaceae*, and other commensals with possible harmful effects on host health, such as *Enterococcus*, *Clostridium* (*sensu stricto*), and *Sarcina*. Many studies have indicated associations between *Erysipelotrichaceae* and host dyslipidemic phenotypes and related metabolic disorders (Zhang et al., 2009; Spencer et al., 2011), and have identified this family as a potential candidate in the etiology and progression of colorectal cancer in the Western world (Candela et al., 2014). On the other hand, incomplete or contradictory information is available on *Sarcina* and its significance in the human gastrointestinal tract. In fact, though it has been reported in cases of gastrointestinal disorders (Ratuapli et al., 2013), *Sarcina* has also been shown to occur frequently in fecal samples of healthy human adults living on principally vegetarian diets in the tropics, namely Uganda and South India, probably as a result of ingestion of contaminated food (Crowther, 1971). In the light of this, the higher abundance of *Sarcina*, we find in the enterocyte-associated microbiota of the Hadza, and especially in women, may be strongly related to their heavily plant-based diet with associated environmental bacteria. This may further be a reflection of the sex-based differences in foraging activities and diet composition, with women spending more time than men digging and snacking tubers (Marlowe, 2001). Of note, the evidence of a sex-related divergence in the epithelial microbiota structure, which was mainly accounted for by *Sarcina* (mean relative abundance, Hadza women, 15.1% vs. men, 2.1%) along with other subdominant components, was exclusive of the Hadza population, as documented for the first time ever in a human group in their gut microbiota (Schnorr et al., 2014). Curiously, *Sarcina* was identified only as a minor component of the Hadza fecal microbiota (mean relative abundance, 0.1%; Schnorr et al., 2014), which seems to suggest specific adhesive properties to enterocytes or a preference for the trophic conditions at the intestinal surface. The same trend was also true for *Enterococcus* (mean relative abundance in feces, <0.01%) and *Clostridium* (0.5%; Schnorr et al., 2014), for which surface proteins with a role in attachment to mucus have already been extensively described (Péchiné et al., 2005; Golińska et al., 2013). Even if the exact role of enterococci and clostridia in human health is still unclear, it is assumed that these microbes establish a close relationship with intestinal cells, exerting a strong influence on gut physiology, metabolism and immunological signaling, contributing to the maintenance of immune function (Atarashi et al., 2011; Wang et al., 2014).

In addition to this, the interaction with the intestinal epithelium also resulted in the selection of a number of minor genera in Hadza compared to Italian samples. This was true for the *Coriobacteriaceae* members *Olsenella* and *Slackia*, for *Barnesiella*, and for the Firmicutes genera *Anaerobacter*, *Melissococcus*, and *Syntrophococcus*. Based on a literature search, we found that *Olsenella* and *Slackia* are regularly isolated from disease sites in the human mouth (e.g., caries, periodontitis, or endodontic infections), but have also been recovered in healthy human and other mammal feces or intestinal samples (Kim et al., 2010; Kraatz et al., 2011). In particular, *Olsenella umbonata* is phenotypically characterized as being well-adapted to the conditions at the absorptive mucosa and is presumably an autochthonous resident of the human gastrointestinal tract (Kraatz et al., 2011). Additionally, some *Slackia* species have attracted the attention of researchers especially for their potent daidzein-to-equol conversion ability (Jin et al., 2010). Considering that the isoflavone daidzein and its glucosides are known to be abundant in legumes, such as soybeans and the roots of other Fabaceae plants (Kaufman et al., 1997), the presence of *Slackia* in the Hadza samples may be related to their diet, which relies heavily on fibrous Fabaceae tubers throughout the year. Consistent with this, equol producer phenotypes are reported to be prevalent among vegetarians, and possibly among those with a low to absent intake of dairy products (Lampe, 2009), which the Hadza also do not consume (Marlowe, 2010). A greater equol production at the intestinal surface could lead to a number of potential benefits for the Hadza health, given the high antioxidant and anti-inflammatory activity of this polyphenol, with protective effects on a variety of diseases (Setchell and Clerici, 2010). Also *Syntrophococcus*, mainly known as a reductive acetogen involved in the degradation of lignin and phenolic acids that abound in plant cell walls (Bernard-Vailhe et al., 1995), may be present as an adaptation to the Hadza foraging lifestyle. Similarly, the exclusive prevalence of *Melissococcus* in the Hadza samples could be related to the consumption of honey-comb (Crittenden, 2011), since *Melissococcus plutonius* is indeed known to be present in honeybee larvae and causes the European foulbrood (Budge et al., 2014).

Unlike the Hadza, the Italian enterocyte-associated microbiota was characterized by SCFA-producing members of Clostridiales Incertae Sedis XIV, *Lachnospiraceae* and *Ruminococcaceae* families, commonly known to be present in a healthy gut and commonly found in mucosa-associated microbiota of Western populations (Eckburg et al., 2005; Hong et al., 2011), supporting the robustness of our experimental model. Within these microbial families, acetate producers, such as *Blautia*, *Dorea*, and *Ruminococcus*, outnumbered those of butyrate (including *Anaerostipes*, *Roseburia*, *Faecalibacterium*, *Oscillibacter*, and *Subdoligranulum*), suggesting a greater availability of acetate at the epithelial interface of Italians. Acetate has recently been shown to improve intestinal defense mediated by epithelial cells, protecting the host against lethal infection (Fukuda et al., 2011), and influence the response of intestinal epithelial cells via innate receptors, dampening the signaling cascades downstream of TLR stimulation (Arpaia, 2014). On the other hand, it is known that acetate serves as a co-substrate to generate butyrate and that this route is prevalent among the human gut colonizers (Flint et al., 2014), which suggests the establishment of a balanced syntrophy at the mucosal surface.

The compositional characteristics of the Hadza epithelial microbiota were reflected in a community functional capability substantially divergent from that of Italians. Besides the high representation of a number of transporters for nutrient acquisition and environment sensing – including the five ABC transporter proteins of the peptides/nickel transport system, recently hypothesized to contribute to gut habitat adaptation through the modulation of surface-exposed molecules (Meehan and Beiko, 2012) – the enterocyte-associated microbiome of the Hadza is enriched in functions that could contribute to the survival in and colonization of the gastrointestinal environment. For instance, compared to Italians, we found greater abundance of proteolytic enzymes, i.e., cysteine peptidase and metalloprotease, which are frequently involved in host-microbe interactions, playing a role in bacterial colonization and sometimes in virulence traits with critical implications for mucosal homeostasis (Pruteanu et al., 2011; Carroll and Maharshak, 2013). Related to this, we also found a reduced abundance of genes for amino acid biosynthesis, especially Trp, suggesting an extensive auxotrophy that is generally at the basis of microbial nutritional strategies for niche adaptation (Sczesnak et al., 2011). These traits reflect the lifestyle of some pathobiont-like specialists that are able to thrive in an inflamed intestinal environment, exploiting the ready nutrient source, including amino acids, provided by host tissue destruction (Morgan et al., 2012).

The Hadza enterocyte-associated microbiome was also enriched in functions generally related to signal transduction, signaling molecules and interaction. In particular, we found a pronounced overrepresentation in the Hadza microbial communities at the intestinal surface of a gene encoding PhzF, an isomerase essential for phenazine synthesis (Blankenfeldt et al., 2004). To date, phenazines are known to serve multiple roles, ranging from modification of cellular redox states to electron shuttling to alternate terminal acceptors, cell signaling, and interestingly, cell adhesion and biofilm formation (Pierson and Pierson, 2010). In this respect, they may be essential for the competitiveness and long-term survival under challenging conditions, as occurs in the multiplicity of different microhabitats and metabolic niches in the mucus layer lining the gut (Macfarlane and Dillon, 2007), thus contributing to the ecological competence of producing strains. The increased abundance of *phzF* gene is likely related to the enrichment in *Pseudomonas*, since phenazines are nitrogen-containing natural products synthesized mostly from soil-dwelling and/or plant-associated *Pseudomonas* species (Pierson and Pierson, 2010). Despite the greater abundance of *Pseudomonas* in the enterocyte adherent microbiota fraction of Hadza compared to Italians (relative abundance, Hadza, 1.1% vs. Italians, 0.5%), any evidence of *Pseudomonas* infection among Hadza who worked with the researchers during collection of fecal samples, was not known or available to the authors. Given that *Pseudomonas* can also be found on rotting fruit and plant material and is a common environmental microbe (Loper et al., 2012), its presence in Hadza stools is likely not surprising but also not informative about potential health-related factors in the context of a healthy luminal microbial environment.

Taken together, our phylogenetic and functional data of the enterocyte-associated microbiome in Hadza therefore suggest the existence of a new mutualistic configuration at the intestinal interface, characterized by the enrichment, compared to urban living Italians, of microorganisms with pathogenic potential. Unlike Italians, Hadza hunter-gatherers maintain a constant direct interface with their environment, and this probably selects for their specific microbiota configuration at the enterocyte surface. We hypothesize that the ability to tolerate the resulting microbe-host cross-talk at the intestinal surface co-evolves through early developmental conditioning, a strategic factor to educate the immune system while preserving homeostasis, which is especially important for maintaining health in a challenging environment without regular access to modern medical care (Lee and Mazmanian, 2010). On the other hand, sanitization, antibiotic usage, and sterile food – typical of Western populations – have probably dissolved the contact with microorganisms with pathogenic potential (Blaser and Falkow, 2009; Kau et al., 2011). This in turn reduces the level of microbe-host cross-talk at the mucosal surface, compromising the ability of our microbiota to hone our immune function, as evidenced by the consequent rapid increase of immunological disorders in the Western world (Ehlers and Kaufmann, 2010).

In summary, through our HT29 cell-based minimal model that simulates the host-microbiota interplay at the intestinal surface, we provide evidence of a peculiar interactive ability of the adherent microbiome that is present in a hunter-gatherer population, supporting the importance of microbiota-host cross-talk along human evolution. However, we deem it important to point out some limitations of our *ex vivo* model, related to both cell line and the use of fecal slurries. Though widely used as one of the best available models for the intestinal epithelium, HT29 cells are indeed recognized as heterogeneous for different aspects, and they usually contain a low proportion of mucus-secreting cells, not fully approximating the intestinal mucosa. On the other side, since the adherent microbiota is assessed from fecal samples, we might have missed some mucosa-associated microorganisms that were not present in the initial fecal microbial community being examined. Further studies are thus needed to gain insight into the dialog at the microbiota-mucosa interface, also from the immunological point of view, in order to unravel the molecular mechanisms underlying the tenuous yet essentially peaceful co-existence between host and microbes in human evolutionary history.

## Author Contributions

MCa, ST, SR, and MCe conceived and designed the experiments; MCe and ST performed fecal slurry-HT29 cell interaction experiments; MF performed HR-SEM analysis; CC, CP, and MSe performed 16S rRNA gene sequencing; ST, MCe, SR, and MSo analyzed the data; ST, MCa, SR, and SS wrote the manuscript; AC, AH, and PB revised and edited the draft. All authors discussed the results, commented on the manuscript and approved the final version.

## Conflict of Interest Statement

The authors declare that the research was conducted in the absence of any commercial or financial relationships that could be construed as a potential conflict of interest.
