# Supplementary material for: Enterocyte-Associated Microbiome of the Hadza Hunter-Gatherers
Source: Front Microbiol. 2016 Jun 6;7:865. doi: 10.3389/fmicb.2016.00865 (PMC4893497; doi:10.3389/fmicb.2016.00865)
Supplement: Supplementary file 1 [file Presentation_1.PDF]

Supplementary Material

Enterocyte-associated microbiome of the Hadza hunter-gatherers

Silvia Turroni*, Simone Rampelli, Manuela Centanni, Stephanie L. Schnorr, Clarissa Consolandi, Marco Severgnini, Clelia Peano, Matteo Soverini, Mirella Falconi, Alyssa N. Crittenden, Amanda G. Henry, Patrizia Brigidi and Marco Candela

***Correspondence:** Silvia Turroni: silvia.turroni@unibo.it

Supplementary Figures and Tables
Supplementary Figures

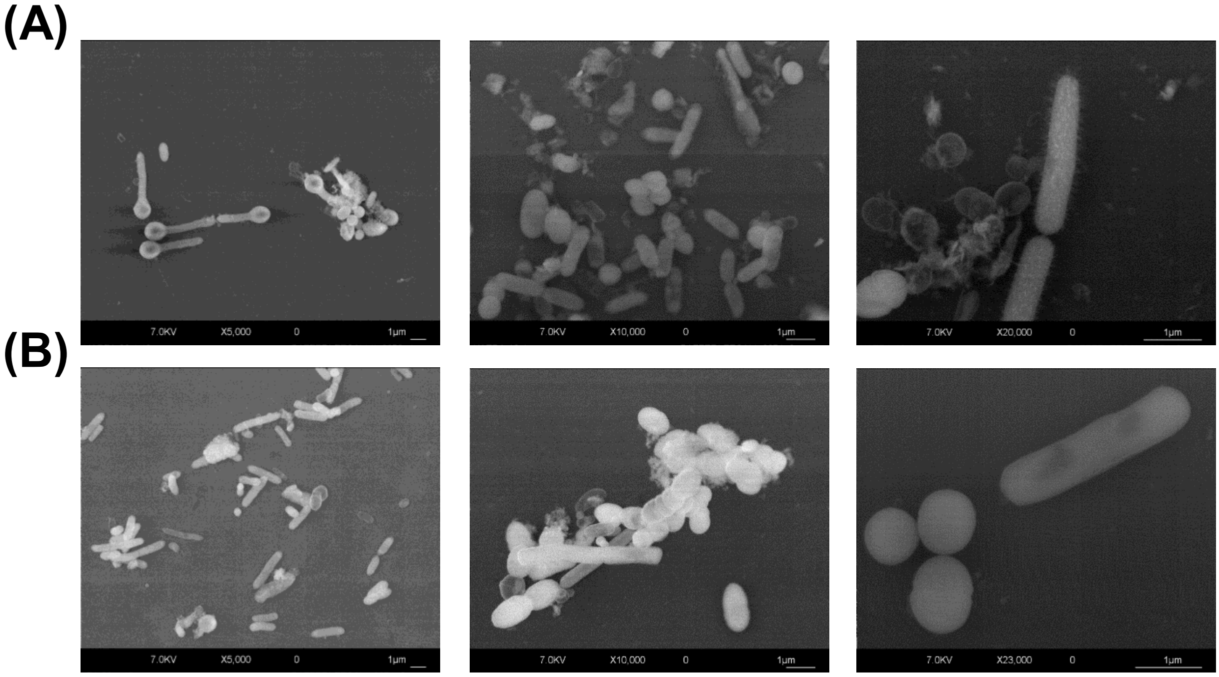

Supplementary Figure 1. High resolution-scanning electron microscopy (HR-SEM) analysis of fecal slurries. The structural integrity of microbial cells in fecal slurries was evaluated by HR-SEM. Representative images for the Hadza (A) and Italians (B) are shown. Magnification and scale bar are indicated for each SEM image.

(A)

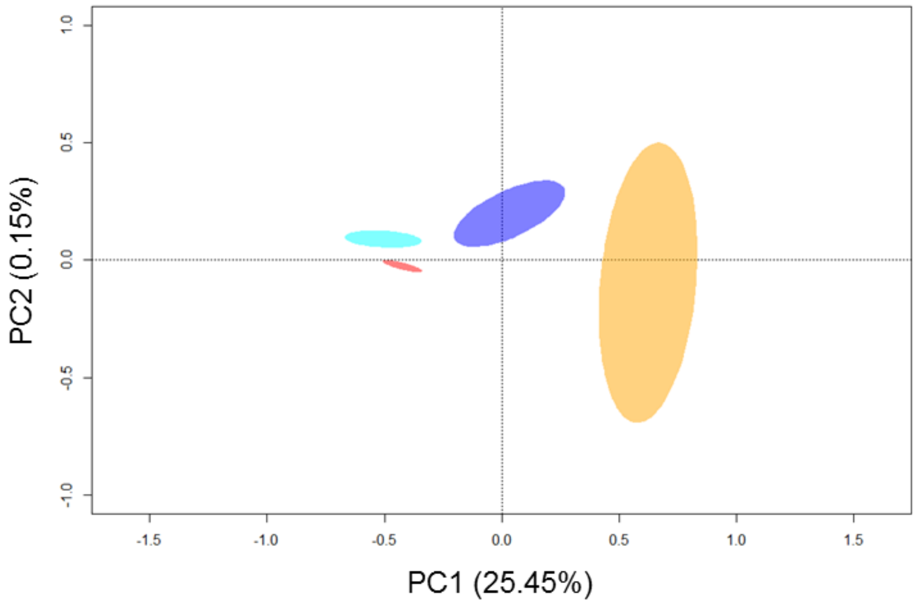
PC2 (0.15%)
PC1 (25.45%)
1.0
0.5
0.0
-0.5
-1.0
-1.5
1.5

(B)

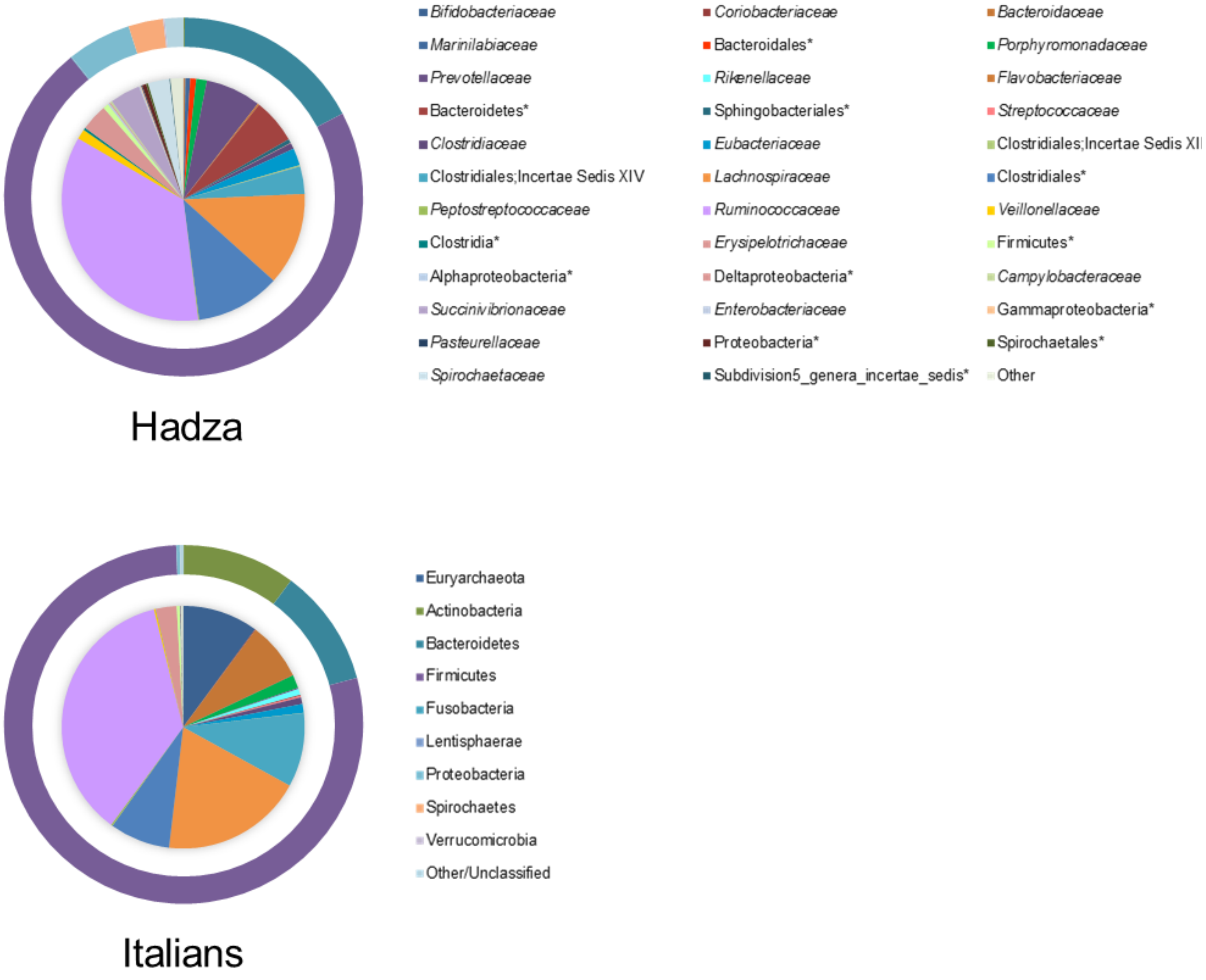
Bifidobacteriaceae
Coriobacteriaceae
Bacteroidaceae
Marinilabiaceae
Bacteroidales*
Porphyromonadaceae
Prevotellaceae
Rikenellaceae
Flavobacteriaceae
Bacteroidetes*
Sphingobacteriales*
Streptococcaceae
Clostridiaceae
Eubacteriaceae
Clostridiales;Incertae Sedis XII
Clostridiales;Incertae Sedis XIV
Lachnospiraceae
Clostridiales*
Peptostreptococcaceae
Ruminococcaceae
Veillonellaceae
Clostridia*
Erysipelotrichaceae
Firmicutes*
Alphaproteobacteria*
Deltaproteobacteria*
Campylobacteraceae
Succinivibrionaceae
Enterobacteriaceae
Gammaproteobacteria*
Pasteurellaceae
Proteobacteria*
Spirochaetales*
Spirochaetaceae
Subdivision5_genera_incertae_sedis*
Other
Hadza
Euryarchaeota
Actinobacteria
Bacteroidetes
Firmicutes
Fusobacteria
Lentisphaerae
Proteobacteria
Spirochaetes
Verrucomicrobia
Other/Unclassified
Italians

Supplementary Figure 2. Comparison between the fecal microbiota and enterocyte-associated microbial communities in Hadza and Italians. (A) PCA of Euclidean distances between genus-level profiles of the fecal microbiota and HT29 cell-associated microbial communities in 21 Hadza and 9 Italians from the study cohort of Schnorr *et al.* (2014). Ellipses include 99% confidence area based on the standard error of the weighted average of sample coordinates. Genera were filtered for those with >0.1% of relative abundance in at least 20% of subjects. Red, the fecal microbiota of Hadza; cyan, the fecal microbiota of Italians; orange, the enterocyte adherent microbiota of Hadza; blue, the enterocyte adherent microbiota of Italians. The two components explain 25.45 and 0.15% of the variance, respectively. A significant separation by population as well as intestinal compartment was found ($P \leq 0.004$, permutation test with pseudo-F ratios). (B). Pie charts summarizing family level taxa in the fecal microbiota of Hadza and Italians (see color legend on the top). Outer rings depict phylum level distribution (see color legend on the bottom). Only families with a mean relative abundance ≥ 0.1% in at least one of the two populations are shown. *denotes unclassified OTU reported at higher taxonomic level.

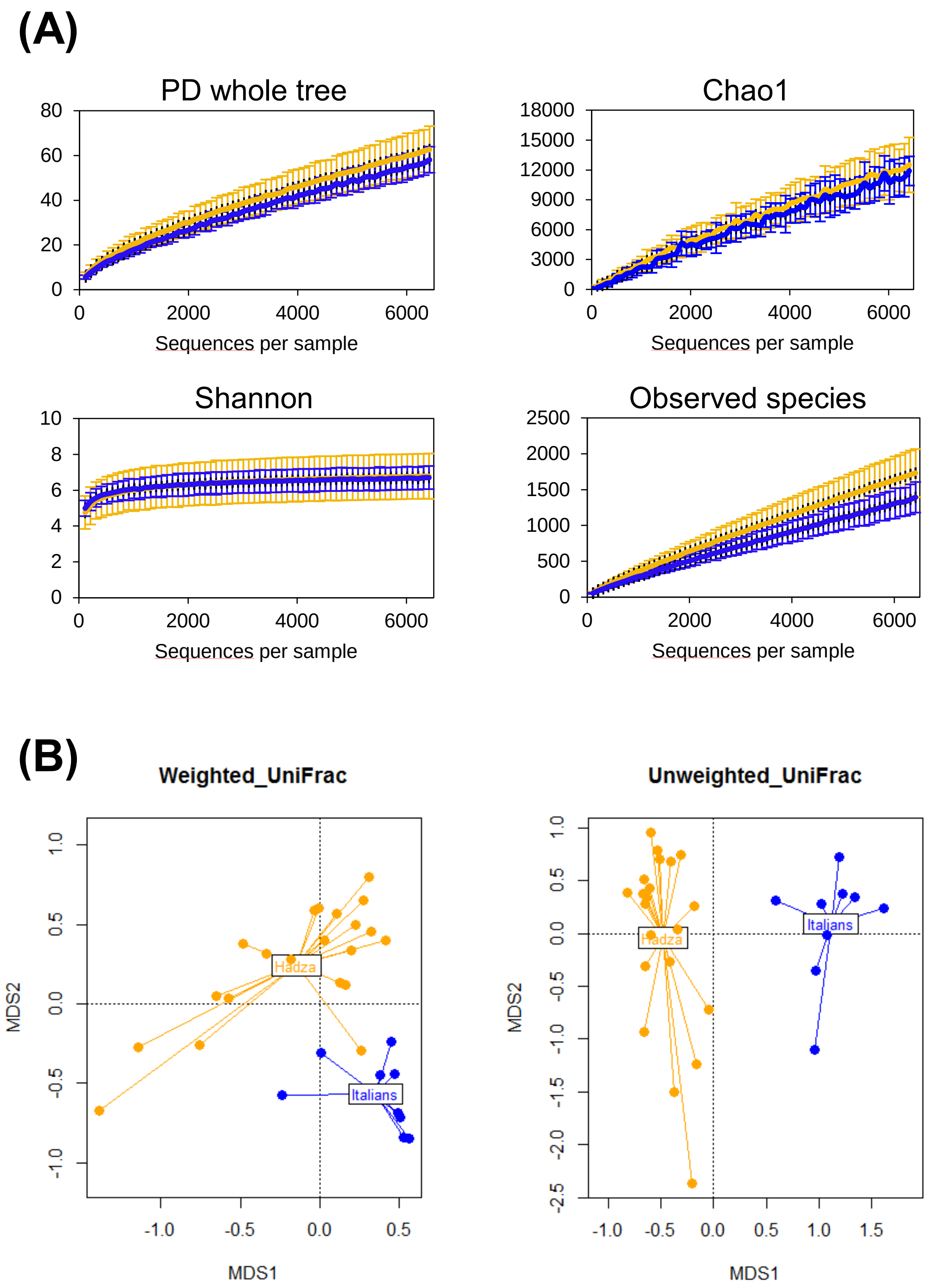

Supplementary Figure 3. Diversity of the enterocyte-associated microbiota in Hadza and Italians. (A). Alpha diversity rarefaction plots. Phylogenetic diversity was estimated by calculating the Faith's phylogenetic diversity index (PD whole tree), Chao1 estimator, Shannon diversity index, and the observed number of species. No significant differences between Hadza and Italians were detected except according to observed species metrics (*P*=0.02, Wilcoxon-Mann-Whitney rank sum test). (B). Weighted and unweighted UniFrac PCoA. The two components explain 56.3 and 11.3% of the variance, respectively. A significant separation in the composition of the enterocyte adherent microbiota between Hadza and Italians was obtained using both metrics (*P*=0.0001, permutation test with pseudo-F ratios). Hadza, orange; Italians, blue.

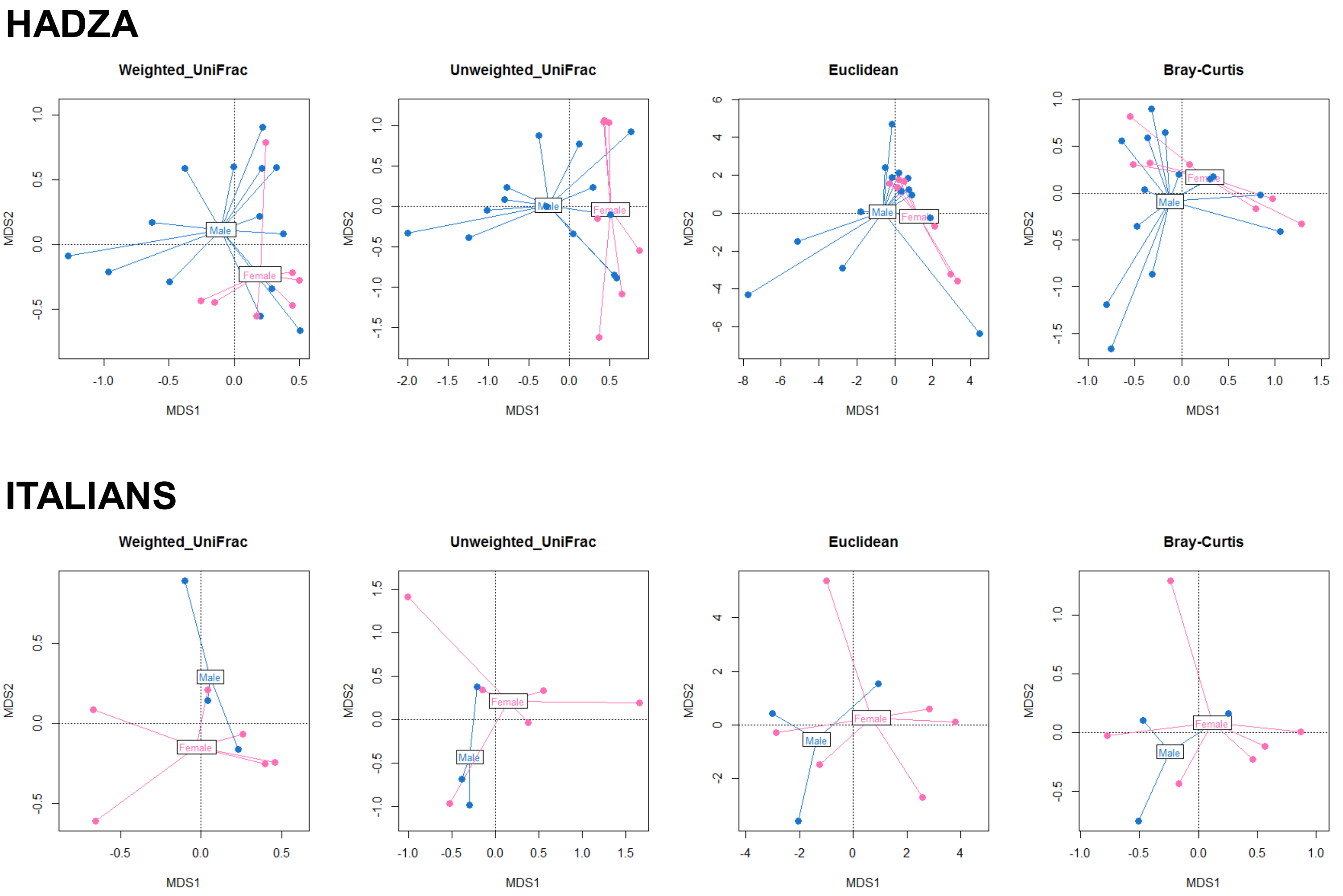

Supplementary Figure 4. Sex difference in the enterocyte-associated microbiota of Hadza and Italians. PCoA based on weighted and unweighted UniFrac, as well as Euclidean and Bray-Curtis distances between relative genus abundance profiles. A significant separation by sex was found only within Hadza cohort according to unweighted UniFrac (*P*=0.03, permutation test with pseudo-F ratios). Females, pink; males, blue.

Supplementary Figure 5. Diversity of PICRUSt-predicted metagenomes.

(A)

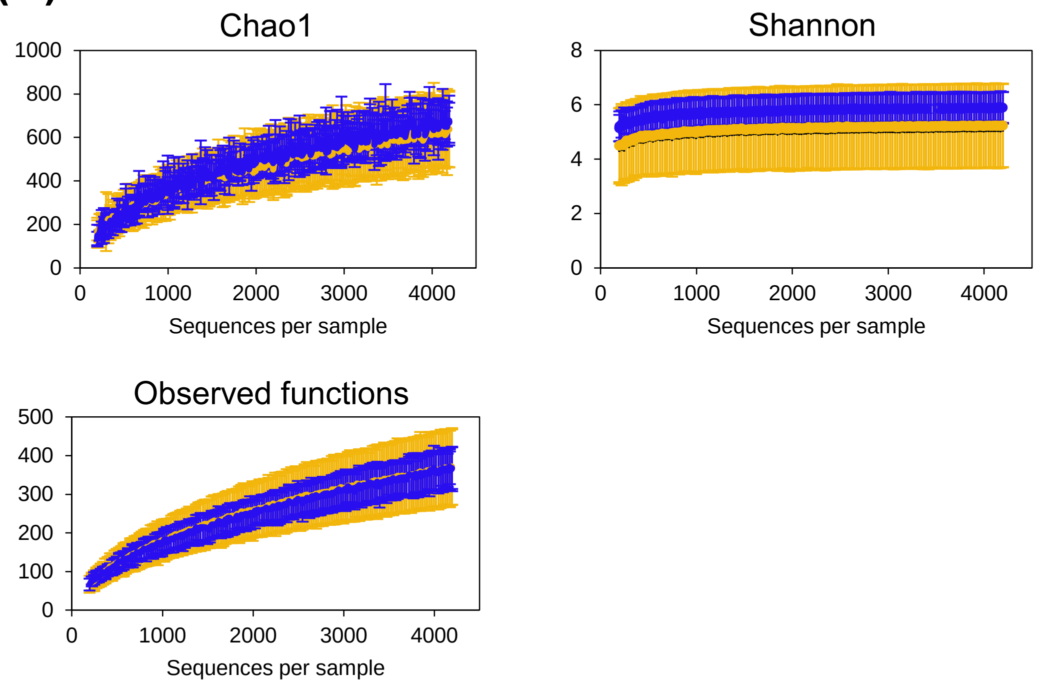

Supplementary Figure 5. Diversity of PICRUSt-predicted metagenomes.

(B)

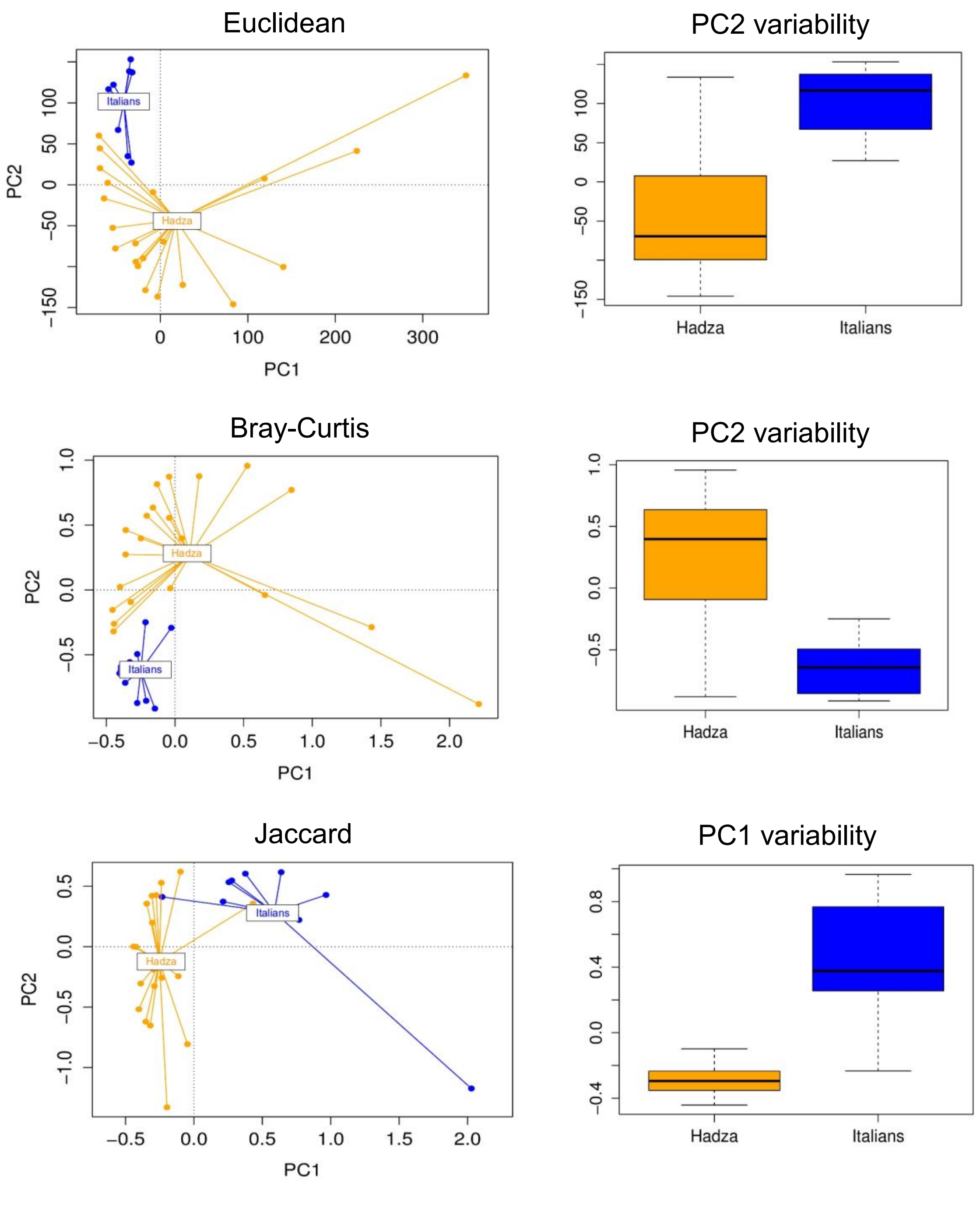

Supplementary Figure 5. Diversity of PICRUSt-predicted metagenomes. (A). Alpha diversity rarefaction plots. Functional diversity was measured using Chao1 estimator, Shannon diversity index, and observed functions. No significant differences between Hadza and Italians were detected except according to Shannon metrics (*P*<0.001, Wilcoxon-Mann-Whitney rank sum test). (B). PCoA based on Euclidean, Bray-Curtis, or Jaccard distances between KEGG ortholog (KO) profiles. The two components explain 86.1%, 84.9% and 80.9% of the variance, respectively. The separation of the functional composition of the enterocyte adherent microbial communities of Hadza and Italians was significant along the first component for Jaccard distances (*P*=0.009,

Wilcoxon-Mann-Whitney rank sum test) and the second component according to Euclidean and Bray-Curtis metrics (*P*=0.02). Hadza, orange; Italians, blue.

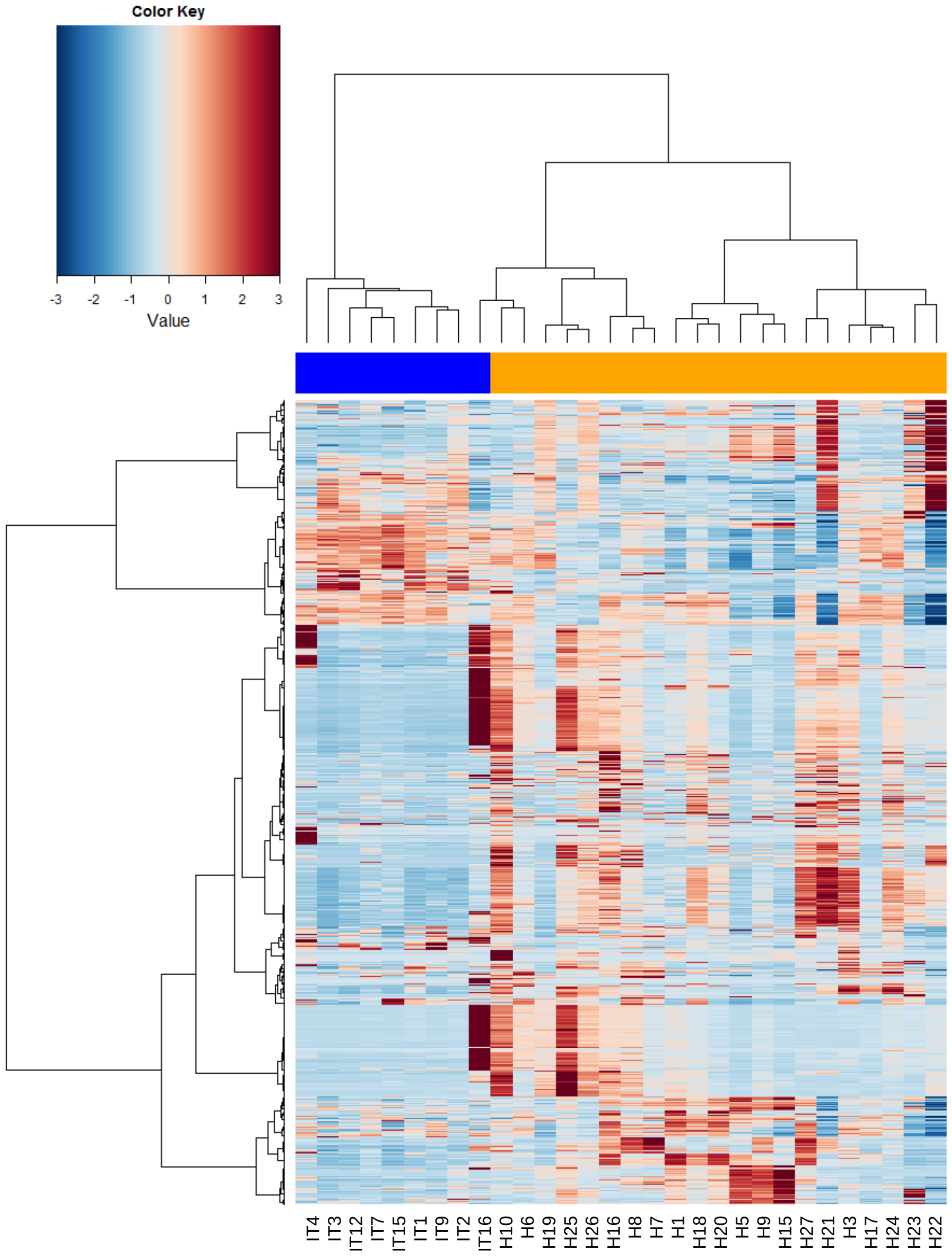

Supplementary Figure 6. KEGG orthologs (KOs) in Hadza and Italian microbial communities at the intestinal epithelial interface. Heat map shows the relative abundances of major KOs (contributing > 0.5% in at least 20% of subjects) calculated using PICRUSt. Hierarchical clustering was performed using the Euclidean distance measure and Ward's linkage method. *P*=1E-6, Fisher's exact test. Hadza, orange; Italians, blue.

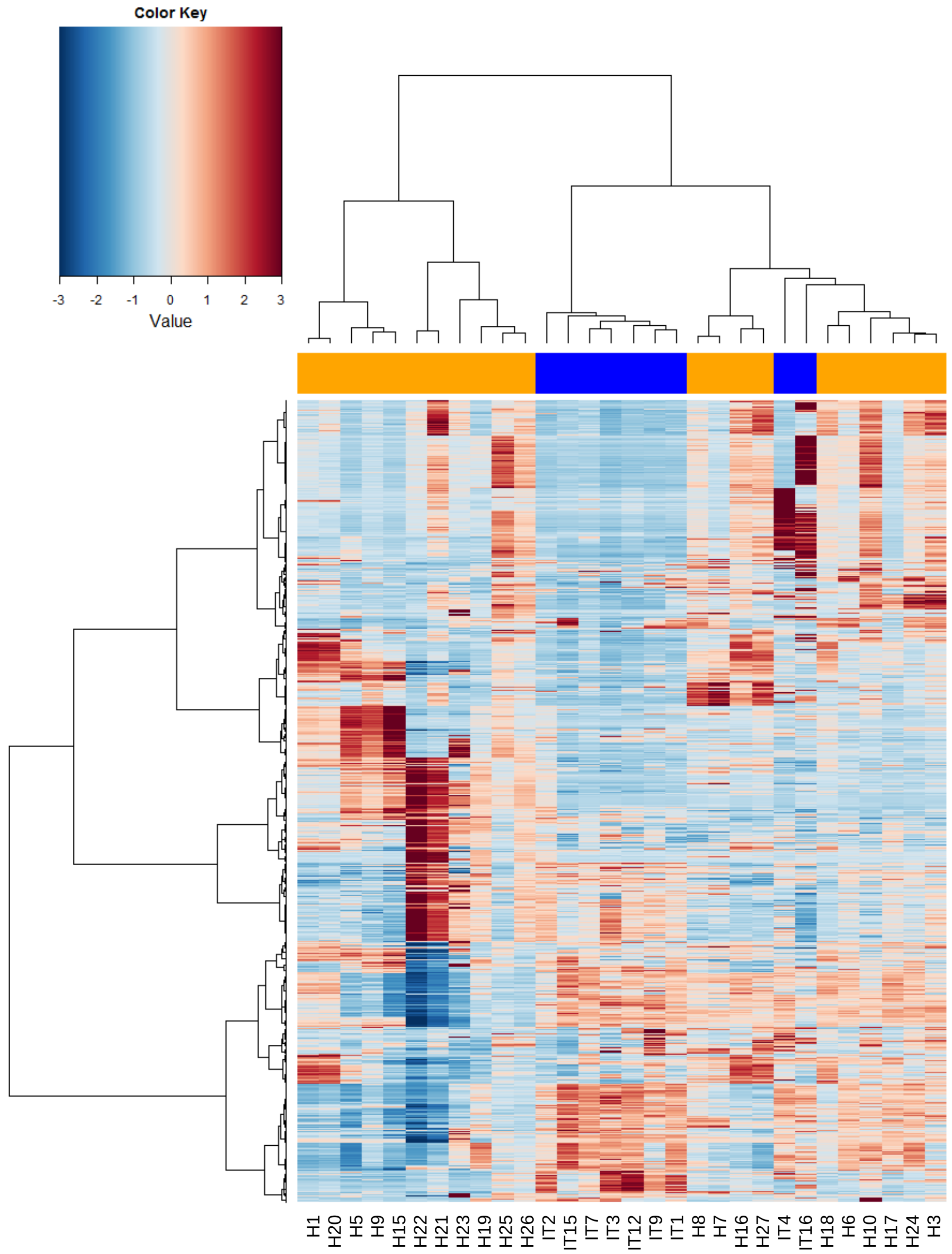

Supplementary Figure 7. Core microbiome at the intestinal mucosa surface. Heat map shows the abundance of 2927 KO identifiers predicted in all the Hadza and Italian samples. Hierarchical clustering was performed using a Spearman's correlation-based dissimilarity metrics and Ward's agglomeration method. No separation was observed between the two populations (*P*>0.05, Fisher's exact test).

Supplementary Tables

Supplementary Table 1. List of differences in relative genus abundance between the enterocyte-associated microbiota of Hadza and Italians

**Genus**	**Hadza**	**Italians**	***P*-value**
Bifidobacterium	0.13	12.19	0.00003
Bifidobacteriaceae unclassified	0.02	0.72	0.00006
Olsenella	0.30	0.02	0.0007
Slackia	0.40	0.09	0.008
Bacteroides	0.22	1.08	0.04
Barnesiella	0.30	0.05	0.01
Prevotella	0.54	0.13	0.0005
Alistipes	0.02	0.17	0.0002
Bacteroidetes unclassified	0.16	0.01	0.0001
Enterococcus	9.92	1.20	0.0002
Melissococcus	0.34	0.04	0.003
Enterococcaceae unclassified	0.64	0.10	0.01
Streptococcus	0.50	0.95	0.01
Anaerobacter	0.68	0.07	0.0008
Clostridium	8.48	0.70	0.001
Clostridiaceae unclassified	0.38	0.11	0.01
Sarcina	6.41	0.01	0.00001
Eubacterium	1.93	0.29	0.00007
Incertae Sedis XIII unclassified	0.12	0.02	0.0001
Blautia	5.95	14.03	0.00004
Anaerostipes	0.33	1.43	0.0002
Coprococcus	2.58	9.45	0.00005
Dorea	1.41	2.78	0.04
Lachnospiraceae unclassified	0.79	2.09	0.00002
Roseburia	0.40	2.24	0.0002
Syntrophococcus	0.23	0.00	0.0008
Oscillibacter	1.25	0.46	0.006
Ruminococcus	0.59	7.45	0.001
Subdoligranulum	0.56	1.76	0.02
Allobaculum	0.16	0.02	0.0002
Bulleidia	0.15	0.00	0.00002
Catenibacterium	1.14	0.02	0.000004
Holdemania	0.17	0.00	0.0005
Erysipelotrichaceae unclassified	0.89	0.04	0.00004
Firmicutes unclassified	0.14	0.02	0.0000003
Bacteria unclassified	0.18	0.08	0.004
Janthinobacterium	0.28	0.14	0.04
Escherichia.Shigella	1.59	0.08	0.003
Enterobacteriaceae unclassified	0.07	0.14	0.04
Pseudomonas	1.10	0.51	0.01
Treponema	0.16	0.00	0.004
Synergistaceae unclassified	0.12	0.01	0.0005

Only significant differences for genera with a mean relative abundance ≥ 0.1% in at least one of the two populations are listed. For each genus, the mean relative abundance (%) and *P*-value of the comparison between Hadza and Italians (Wilcoxon-Mann-Whiteny rank sum test) are reported.

Supplementary Table 2. List of differences in relative genus abundance between the enterocyte-associated microbiota of Hadza men and women

**Genus**	**Men**	**Women**	***P*-value**
Slackia	0.53	0.15	0.01
Pediococcus	0.13	0.00	0.04
Sarcina	2.05	15.12	0.04
Eubacterium	2.40	1.01	0.03
Incertae Sedis XIII unclassified	0.15	0.06	0.03
Oribacterium	0.04	0.11	0.03
Treponema	0.06	0.34	0.01

Only significant differences for genera with a mean relative abundance ≥ 0.1% in Hadza men or women are listed. For each genus, the mean relative abundance (%) and *P*-value of the comparison between men and women (Wilcoxon-Mann-Whiteny rank sum test) are reported.

Supplementary Table 3. Top 100 KO identifiers most represented in the enterocyte-associated microbiome of Hadza

KO ID	Name	Definition	Pathway	Module
K06147	ABCB-BAC	ATP-binding cassette, subfamily B, bacterial		
K02003	ABC.CD.A	putative ABC transport system ATP-binding protein		M00258 Putative ABC transport system
K02025	ABC.MS.P	multiple sugar transport system permease protein		M00207 Putative multiple sugar transport system
K03088	SIG3.2, rpoE	RNA polymerase sigma-70 factor, ECF subfamily		
K02026	ABC.MS.P1	multiple sugar transport system permease protein		M00207 Putative multiple sugar transport system
K02004	ABC.CD.P	putative ABC transport system permease protein		M00258 Putative ABC transport system
K09687		antibiotic transport system ATP-binding protein		
K07024				
K02529	lacI, galR	LacI family transcriptional regulator		
K02027	ABC.MS.S	multiple sugar transport system substrate-binding protein		M00207 Putative multiple sugar transport system
K01990	ABC-2.A	ABC-2 type transport system ATP-binding protein		M00254 ABC-2 type transport system
K02006	cbiO	cobalt/nickel transport system ATP-binding protein	ko02010 ABC transporters	M00245 Cobalt/nickel transport system<br>M00246 Nickel transport system
K02035	ABC.PE.S	peptide/nickel transport system substrate-binding protein		M00239 Peptides/nickel transport system
K01834	PGAM, gpmA	2,3-bisphosphoglycerate-dependent phosphoglycerate mutase [EC:5.4.2.11]	ko00010 Glycolysis / Gluconeogenesis<br>ko00260 Glycine, serine and threonine metabolism<br>ko00680 Methane metabolism<br>ko01200 Carbon metabolism<br>ko01230 Biosynthesis of amino acids<br>ko05230 Central carbon metabolism in cancer	M00001 Glycolysis (Embden-Meyerhof pathway), glucose => pyruvate<br>M00002 Glycolysis, core module involving three-carbon compounds<br>M00003 Gluconeogenesis, oxaloacetate => fructose-6P
K02030	ABC.PA.S	polar amino acid transport system substrate-binding protein		M00236 Putative polar amino acid transport system
K00936	E2.7.3.-	Phosphotransferases with a nitrogenous group as acceptor		
K03406	mcp	methyl-accepting chemotaxis protein	ko02020 Two-component system<br>ko02030 Bacterial chemotaxis	
K02057	ABC.SS.P	simple sugar transport system permease protein		M00221 Putative simple sugar transport system
K03091	SIG3.4	RNA polymerase sporulation-specific sigma factor		
K03496	parA, soj	chromosome partitioning protein		
K13789	GGPS	geranylgeranyl diphosphate synthase, type II [EC:2.5.1.1 2.5.1.10 2.5.1.29]	ko00900 Terpenoid backbone biosynthesis	M00364 C10-C20 isoprenoid biosynthesis, bacteria<br>M00366 C10-C20 isoprenoid biosynthesis, plants
K00266	gltD	glutamate synthase (NADPH/NADH) small chain [EC:1.4.1.13 1.4.1.14]	ko00250 Alanine, aspartate and glutamate metabolism<br>ko00910 Nitrogen metabolism<br>ko01230 Biosynthesis of amino acids	
K09686		antibiotic transport system permease protein		
K06180	rluD	23S rRNA pseudouridine1911/1915/1917 synthase [EC:5.4.99.23]		
K00974	cca	tRNA nucleotidyltransferase (CCA-adding enzyme) [EC:2.7.7.72 3.1.3.- 3.1.4.-]	ko03013 RNA transport	
K02008	cbiQ	cobalt/nickel transport system permease protein	ko02010 ABC transporters	M00245 Cobalt/nickel transport system<br>M00246 Nickel transport system
K01534	zntA	Cd2+/Zn2+-exporting ATPase [EC:3.6.3.3 3.6.3.5]		
K03497	parB, spo0J	chromosome partitioning protein, ParB family		
K07258	dacC, dacA, dacD	D-alanyl-D-alanine carboxypeptidase (penicillin-binding protein 5/6) [EC:3.4.16.4]	ko00550 Peptidoglycan biosynthesis	
K11754	folC	dihydrofolate synthase / folylpolyglutamate synthase [EC:6.3.2.12 6.3.2.17]	ko00790 Folate biosynthesis	M00126 Tetrahydrofolate biosynthesis, GTP => THF
K00599	METTL6	methyltransferase-like protein 6 [EC:2.1.1.-]		
K03657	uvrD, pcrA	DNA helicase II / ATP-dependent DNA helicase PcrA [EC:3.6.4.12]	ko03420 Nucleotide excision repair<br>ko03430 Mismatch repair	
K01104	E3.1.3.48	protein-tyrosine phosphatase [EC:3.1.3.48]		
K00615	E2.2.1.1, tktA, tktB	transketolase [EC:2.2.1.1]	ko00030 Pentose phosphate pathway<br>ko00710 Carbon fixation in photosynthetic organisms<br>ko01051 Biosynthesis of ansamycins<br>ko01200 Carbon metabolism<br>ko01230 Biosynthesis of amino acids	M00004 Pentose phosphate pathway (Pentose phosphate cycle)<br>M00007 Pentose phosphate pathway, non-oxidative phase, fructose 6P => ribose 5P<br>M00165 Reductive pentose phosphate cycle (Calvin cycle)<br>M00167 Reductive pentose phosphate cycle, glyceraldehyde-3P => ribulose-5P
K02028	ABC.PA.A	polar amino acid transport system ATP-binding protein [EC:3.6.3.21]		M00236 Putative polar amino acid transport system
K00850	pfkA, PFK	6-phosphofructokinase 1 [EC:2.7.1.11]	ko00010 Glycolysis / Gluconeogenesis<br>ko00030 Pentose phosphate pathway<br>ko00051 Fructose and mannose metabolism<br>ko00052 Galactose metabolism<br>ko00680 Methane metabolism<br>ko01200 Carbon metabolism<br>ko01230 Biosynthesis of amino acids<br>ko03018 RNA degradation<br>ko04152 AMPK signaling pathway<br>ko05230 Central carbon metabolism in cancer	M00001 Glycolysis (Embden-Meyerhof pathway), glucose => pyruvate<br>M00345 Formaldehyde assimilation, ribulose monophosphate pathway
K00059	fabG	3-oxoacyl-[acyl-carrier protein] reductase [EC:1.1.1.100]	ko00061 Fatty acid biosynthesis<br>ko00780 Biotin metabolism	M00083 Fatty acid biosynthesis, elongation<br>M00572 Pimeloyl-ACP biosynthesis, BioC-BioH pathway, malonyl-ACP => pimeloyl-ACP

			ko01040 Biosynthesis of unsaturated fatty acids<br>ko01212 Fatty acid metabolism	
K10439	rbsB	ribose transport system substrate-binding protein	ko02010 ABC transporters<br>ko02030 Bacterial chemotaxis	M00212 Ribose transport system
K02033	ABC.PE.P	peptide/nickel transport system permease protein		M00239 Peptides/nickel transport system
K01992	ABC-2.P	ABC-2 type transport system permease protein		M00254 ABC-2 type transport system
K03100	lepB	signal peptidase I [EC:3.4.21.89]	ko03060 Protein export	
K02029	ABC.PA.P	polar amino acid transport system permease protein		M00236 Putative polar amino acid transport system
K02015	ABC.FEV.P	iron complex transport system permease protein	ko02010 ABC transporters	M00240 Iron complex transport system
K06148	ABCC-BAC	ATP-binding cassette, subfamily C, bacterial	ko02010 ABC transporters	
K02034	ABC.PE.P1	peptide/nickel transport system permease protein		M00239 Peptides/nickel transport system
K07720	yesN	two-component system, response regulator YesN	ko02020 Two-component system	M00519 YesM-YesN two-component regulatory system
K07718	yesM	two-component system, sensor histidine kinase YesM [EC:2.7.13.3]	ko02020 Two-component system	M00519 YesM-YesN two-component regulatory system
K03111	ssb	single-strand DNA-binding protein	ko03030 DNA replication<br>ko03430 Mismatch repair<br>ko03440 Homologous recombination	
K02032	ABC.PE.A1	peptide/nickel transport system ATP-binding protein		M00239 Peptides/nickel transport system
K02078	acp	acyl carrier protein		
K00384	trxB	thioredoxin reductase (NADPH) [EC:1.8.1.9]	ko00240 Pyrimidine metabolism<br>ko00450 Selenocompound metabolism	
K04758	feoA	ferrous iron transport protein A		
K01491	folD	methylenetetrahydrofolate dehydrogenase (NADP+) / methenyltetrahydrofolate cyclohydrolase [EC:1.5.1.5 3.5.4.9]	ko00670 One carbon pool by folate<br>ko00720 Carbon fixation pathways in prokaryotes<br>ko01200 Carbon metabolism	M00140 C1-unit interconversion, prokaryotes<br>M00377 Reductive acetyl-CoA pathway (Wood-Ljungdahl pathway)
K03671	trxA	thioredoxin 1		
K00975	glgC	glucose-1-phosphate adenylyltransferase [EC:2.7.7.27]	ko00500 Starch and sucrose metabolism<br>ko00520 Amino sugar and nucleotide sugar metabolism	M00565 Trehalose biosynthesis, D-glucose 1P => trehalose
K01091	E3.1.3.18, gph	phosphoglycolate phosphatase [EC:3.1.3.18]	ko00630 Glyoxylate and dicarboxylate metabolism	M00532 Photorespiration
K01961	accC	acetyl-CoA carboxylase, biotin carboxylase subunit [EC:6.4.1.2 6.3.4.14]	ko00061 Fatty acid biosynthesis<br>ko00253 Tetracycline biosynthesis<br>ko00620 Pyruvate metabolism<br>ko00640 Propanoate metabolism<br>ko00720 Carbon fixation pathways in prokaryotes<br>ko01200 Carbon metabolism<br>ko01212 Fatty acid metabolism	M00082 Fatty acid biosynthesis, initiation<br>M00376 3-Hydroxypropionate bi-cycle
K01915	glnA, GLUL	glutamine synthetase [EC:6.3.1.2]	ko00250 Alanine, aspartate and glutamate metabolism<br>ko00330 Arginine and proline metabolism<br>ko00630 Glyoxylate and dicarboxylate metabolism<br>ko00910 Nitrogen metabolism<br>ko01230 Biosynthesis of amino acids<br>ko02020 Two-component system<br>ko04724 Glutamatergic synapse<br>ko04727 GABAergic synapse	
K03686	dnaJ	molecular chaperone DnaJ		
K02794	PTS-Man_EIIB, manX	PTS system, mannose-specific IIB component [EC:2.7.1.69]	ko00051 Fructose and mannose metabolism<br>ko00520 Amino sugar and nucleotide sugar metabolism<br>ko02060 Phosphotransferase system (PTS)	M00276 PTS system, mannose-specific II component
K00945	cmk	cytidylate kinase [EC:2.7.4.14]	ko00240 Pyrimidine metabolism	M00052 Pyrimidine ribonucleotide biosynthesis, UMP => UDP/UTP,CDP/CTP
K02031	ABC.PE.A	peptide/nickel transport system ATP-binding protein		M00239 Peptides/nickel transport system
K04487	iscS, NFS1	cysteine desulfurase [EC:2.8.1.7]	ko00730 Thiamine metabolism<br>ko04122 Sulfur relay system	
K03310	TC.AGCS	alanine or glycine:cation symporter, AGCS family		
K07483		transposase		
K06950		uncharacterized protein		
K02050	ABC.SN.P	NitT/TauT family transport system permease protein		M00188 NitT/TauT family transport system
K07133				
K03798	ftsH, hflB	cell division protease FtsH [EC:3.4.24.-]		
K11753	ribF	riboflavin kinase / FMN adenylyltransferase [EC:2.7.1.26 2.7.7.2]	ko00740 Riboflavin metabolism	M00125 Riboflavin biosynthesis, GTP => riboflavin/FMN/FAD
K05366	mrcA	penicillin-binding protein 1A [EC:2.4.1.- 3.4.-.-]	ko00550 Peptidoglycan biosynthesis<br>ko01501 beta-Lactam resistance	
K01625	eda	2-dehydro-3-deoxyphosphogluconate aldolase / (4S)-4-hydroxy-2-oxoglutarate aldolase [EC:4.1.2.14 4.1.3.42]	ko00030 Pentose phosphate pathway<br>ko00630 Glyoxylate and dicarboxylate metabolism<br>ko01200 Carbon metabolism	M00008 Entner-Doudoroff pathway, glucose-6P => glyceraldehyde-3P + pyruvate<br>M00061 D-Glucuronate degradation, D-glucuronate => pyruvate + D-glyceraldehyde 3P<br>M00308 Semi-phosphorylative Entner-Doudoroff pathway, gluconate => glycerate-3P<br>M00631D-Galacturonate degradation (bacteria), D-galacturonate => pyruvate + D-glyceraldehyde 3P
K03499	trkA	trk system potassium uptake protein TrkA		
K01462	PDF, def	peptide deformylase [EC:3.5.1.88]		

K03308	TC.NSS	neurotransmitter:Na+ symporter, NSS family		
K02761	PTS-Cel-EIIC, celB	PTS system, cellobiose-specific IIC component	ko02060 Phosphotransferase system (PTS)	M00275 PTS system, cellobiose-specific II component
K04069	pflA, pflC, pflE	pyruvate formate lyase activating enzyme [EC:1.97.1.4]		
K02016	ABC.FEV.S	iron complex transport system substrate-binding protein	ko02010 ABC transporters	M00240 Iron complex transport system
K03169	topB	DNA topoisomerase III [EC:5.99.1.2]		
K01703	leuC	3-isopropylmalate/(R)-2-methylmalate dehydratase large subunit [EC:4.2.1.33 4.2.1.35]	ko00290 Valine, leucine and isoleucine biosynthesis<br>ko00660 C5-Branched dibasic acid metabolism<br>ko01210 2-Oxocarboxylic acid metabolism<br>ko01230 Biosynthesis of amino acids	M00432 Leucine biosynthesis, 2-oxoisovalerate => 2-oxoisocaproate<br>M00535 Isoleucine biosynthesis, pyruvate => 2-oxobutanoate
K11189	PTS-HPR	phosphocarrier protein		
K00540		oxidoreductase		
K01704	leuD	3-isopropylmalate/(R)-2-methylmalate dehydratase small subunit [EC:4.2.1.33 4.2.1.35]	ko00290 Valine, leucine and isoleucine biosynthesis<br>ko00660 C5-Branched dibasic acid metabolism<br>ko01210 2-Oxocarboxylic acid metabolism<br>ko01230 Biosynthesis of amino acids	M00432 Leucine biosynthesis, 2-oxoisovalerate => 2-oxoisocaproate<br>M00535 Isoleucine biosynthesis, pyruvate => 2-oxobutanoate
K06023	hprK, ptsK	HPr kinase/phosphorylase [EC:2.7.11.- 2.7.4.-]		
K06889				
K07088				
K07029				
K01223	E3.2.1.86B, bglA	6-phospho-beta-glucosidase [EC:3.2.1.86]	ko00010 Glycolysis / Gluconeogenesis	
K01752	E4.3.1.17, sdaA	L-serine dehydratase [EC:4.3.1.17]	ko00260 Glycine, serine and threonine metabolism<br>ko00270 Cysteine and methionine metabolism<br>ko01200 Carbon metabolism<br>ko01230 Biosynthesis of amino acids	
K03498	trkH	trk system potassium uptake protein TrkH		
K07171	mazF, ndoA, chpA	mRNA interferase MazF [EC:3.1.-.-]		
K02795	PTS-Man-EIIC, manY	PTS system, mannose-specific IIC component	ko00051 Fructose and mannose metabolism<br>ko00520 Amino sugar and nucleotide sugar metabolism<br>ko02060 Phosphotransferase system (PTS)	M00276 PTS system, mannose-specific II component
K00602	purH	phosphoribosylaminoimidazolecarboxamide formyltransferase / IMP cyclohydrolase [EC:2.1.2.3 3.5.4.10]	ko00230 Purine metabolism<br>ko00670 One carbon pool by folate	M00048 Inosine monophosphate biosynthesis, PRPP + glutamine => IMP
K07979	ytrA	GntR family transcriptional regulator		
K05349	bglX	beta-glucosidase [EC:3.2.1.21]	ko00460 Cyanoamino acid metabolism<br>ko00500 Starch and sucrose metabolism<br>ko00940 Phenylpropanoid biosynthesis	
K01190	lacZ	beta-galactosidase [EC:3.2.1.23]	ko00052 Galactose metabolism<br>ko00511 Other glycan degradation<br>ko00600 Sphingolipid metabolism	
K01714	dapA	4-hydroxy-tetrahydrodipicolinate synthase [EC:4.3.3.7]	ko00300 Lysine biosynthesis<br>ko01230 Biosynthesis of amino acids	M00016 Lysine biosynthesis, succinyl-DAP pathway, aspartate => lysine<br>M00525 Lysine biosynthesis, acetyl-DAP pathway, aspartate => lysine<br>M00526 Lysine biosynthesis, DAP dehydrogenase pathway, aspartate => lysine<br>M00527 Lysine biosynthesis, DAP aminotransferase pathway, aspartate => lysine
K02355	fusA, GFM, EFG	elongation factor G		
K02013	ABC.FEV.A	iron complex transport system ATP-binding protein [EC:3.6.3.34]	ko02010 ABC transporters	M00240 Iron complex transport system
K03205	virD4, lvhD4	type IV secretion system protein VirD4	ko03070 Bacterial secretion system	M00333 Type IV secretion system

Where available, information on KEGG pathways and/or modules is provided.

Supplementary Table 4. List of differences in the proportion of KEGG pathways between the predicted enterocyte-associated metagenome of Hadza and Italians

**KEGG pathway**	**Hadza**	**Italians**	***P*-value**
**Metabolism**			
Amino acid metabolism	9.06	9.94	0.0001
Biosynthesis of other secondary metabolites	0.79	0.88	0.0001
Energy metabolism	5.60	5.94	0.002
Metabolism of cofactors and vitamins	3.93	4.37	0.000009
**Genetic Information Processing**			
Folding, sorting and degradation	2.25	2.34	0.002
**Environmental Information Processing**			
Membrane Transport	14.46	13.89	0.02
Signal Transduction	1.59	1.41	0.004
Signalling Molecules and Interaction	0.18	0.14	0.001
**Cellular Processes**			
Cell Growth and Death	0.51	0.53	0.009
Cell Motility	1.94	1.53	0.03
**Human Diseases**			
Infectious Diseases	0.39	0.35	0.0004
**Unclassified**			
Metabolism	2.52	2.33	0.002
Poorly Characterized	4.89	4.76	0.001

Only significant differences for KEGG pathways with a mean proportion ≥ 0.1% in at least one of the two populations are listed. For each pathway, the mean proportion (%) and *P*-value of the comparison between Hadza and Italians (Wilcoxon-Mann-Whiteny rank sum test) are reported.

Supplementary Table 5. List of KO identifiers significantly differentially abundant between the enterocyte-associated microbiome of Hadza and Italians

KO ID	Name	Definition	Pathway	Module	Mean Hadza	Mean Italians	*P* -value
K02025	ABC.MS.P	multiple sugar transport system permease protein		M00207 Putative multiple sugar transport system	10119.24	14502.22	0.01
K02026	ABC.MS.P1	multiple sugar transport system permease protein		M00207 Putative multiple sugar transport system	9544.00	13358.33	0.02
K05349	bglX	beta-glucosidase [EC:3.2.1.21]	ko00460 Cyanoamino acid metabolism<br>ko00500 Starch and sucrose metabolism<br>ko00940 Phenylpropanoid biosynthesis		2705.62	4438.00	0.0004
K01190	lacZ	beta-galactosidase [EC:3.2.1.23]	ko00052 Galactose metabolism<br>ko00511 Other glycan degradation<br>ko00600 Sphingolipid metabolism		2691.86	3621.00	0.002
K01652	E2.2.1.6L, ilvB, ilvG, ilvI	acetolactate synthase I/II/III large subunit [EC:2.2.1.6]	ko00290 Valine, leucine and isoleucine biosynthesis<br>ko00650 Butanoate metabolism<br>ko00660 C5-Branched dibasic acid metabolism<br>ko00770 Pantothenate and CoA biosynthesis<br>ko01210 2-Oxocarboxylic acid metabolism<br>ko01230 Biosynthesis of amino acids	M00019 Valine/isoleucine biosynthesis, pyruvate => valine / 2-oxobutanoate => isoleucine<br>M00570 Isoleucine biosynthesis, threonine => 2-oxobutanoate => isoleucine	2487.38	3146.67	0.005
K02647	cdaR	carbohydrate diacid regulator			1699.71	2343.22	0.03
K01710	E4.2.1.46, rfbB, rffG	dTDP-glucose 4,6-dehydratase [EC:4.2.1.46]	ko00521 Streptomycin biosynthesis<br>ko00523 Polyketide sugar unit biosynthesis<br>ko01055 Biosynthesis of vancomycin group antibiotics		1615.52	2162.78	0.01
K01182	E3.2.1.10	oligo-1,6-glucosidase [EC:3.2.1.10]	ko00052 Galactose metabolism<br>ko00500 Starch and sucrose metabolism		1352.05	2562.33	0.02
K07407	E3.2.1.22B, galA, rafA	alpha-galactosidase [EC:3.2.1.22]	ko00052 Galactose metabolism<br>ko00561 Glycerolipid metabolism<br>ko00600 Sphingolipid metabolism<br>ko00603 Glycosphingolipid biosynthesis - globo series		1507.76	2177.89	0.008
K00705	malQ	4-alpha-glucanotransferase [EC:2.4.1.25]	ko00500 Starch and sucrose metabolism		1475.81	2138.33	0.02
K02760	PTS-Cel-EIIB, celA	PTS system, cellobiose-specific IIB component [EC:2.7.1.69]	ko02060 Phosphotransferase system (PTS)	M00275 PTS system, cellobiose-specific II component	2079.29	668.11	0.003
K12308	bgaB, lacA	beta-galactosidase [EC:3.2.1.23]	ko00052 Galactose metabolism		1219.38	2236.67	0.003
K01626	E2.5.1.54, aroF, aroG, aroH	3-deoxy-7-phosphoheptulonate synthase [EC:2.5.1.54]	ko00400 Phenylalanine, tyrosine and tryptophan biosynthesis<br>ko01230 Biosynthesis of amino acids	M00022 Shikimate pathway, phosphoenolpyruvate + erythrose-4P => chorismate	1037.19	1858.11	0.002
K01809	manA, MPI	mannose-6-phosphate isomerase [EC:5.3.1.8]	ko00051 Fructose and mannose metabolism<br>ko00520 Amino sugar and nucleotide sugar metabolism	M00114 Ascorbate biosynthesis, plants, glucose-6P => ascorbate	1292.86	943.22	0.03
K03154	thiS	sulfur carrier protein	ko04122 Sulfur relay system		910.71	1706.00	0.002
K09691	ABC-2.LPSE.A	lipopolysaccharide transport system ATP-binding protein	ko02010 ABC transporters	M00250 Lipopolysaccharide transport system	930.71	1659.33	0.005
K02438	treX, glgX	glycogen operon protein [EC:3.2.1.-]	ko00500 Starch and sucrose metabolism	M00565 Trehalose biosynthesis, D-glucose 1P => trehalose	841.62	1820.56	0.0004
K01696	trpB	tryptophan synthase beta chain [EC:4.2.1.20]	ko00260 Glycine, serine and threonine metabolism<br>ko00400 Phenylalanine, tyrosine and tryptophan biosynthesis<br>ko01230 Biosynthesis of amino acids	M00023 Tryptophan biosynthesis, chorismate => tryptophan	956.90	1423.89	0.003
K02919	RP-L36, MRPL36, rpmJ	large subunit ribosomal protein L36	ko03010 Ribosome	M00178 Ribosome, bacteria	1197.19	851.67	0.005
K01209	E3.2.1.55, abfA	alpha-N-arabinofuranosidase [EC:3.2.1.55]	ko00520 Amino sugar and nucleotide sugar metabolism		807.43	1697.44	0.0004
K02759	PTS-Cel-EIIA, celC	PTS system, cellobiose-specific IIA component [EC:2.7.1.69]	ko02060 Phosphotransferase system (PTS)	M00275 PTS system, cellobiose-specific II component	1333.00	438.00	0.0008
K00765	hisG	ATP phosphoribosyltransferase [EC:2.4.2.17]	ko00340 Histidine metabolism<br>ko01230 Biosynthesis of amino acids	M00026 Histidine biosynthesis, PRPP => histidine	897.14	1419.11	0.01
K00244	frdA	fumarate reductase flavoprotein subunit [EC:1.3.5.4]	ko00020 Citrate cycle (TCA cycle)<br>ko00190 Oxidative phosphorylation<br>ko00620 Pyruvate metabolism<br>ko00650 Butanoate metabolism<br>ko00720 Carbon fixation pathways in prokaryotes<br>ko01200 Carbon metabolism<br>ko02020 Two-component system	M00009 Citrate cycle (TCA cycle, Krebs cycle)<br>M00011 Citrate cycle, second carbon oxidation, 2-oxoglutarate => oxaloacetate<br>M00150 Fumarate reductase, prokaryotes<br>M00173 Reductive citrate cycle (Arnon-Buchanan cycle)	1315.24	332.56	0.04
K03147	thiC	phosphomethylpyrimidine synthase [EC:4.1.99.17]	ko00730 Thiamine metabolism	M00127 Thiamine biosynthesis, AIR => thiamine-P/thiamine-2P	877.95	1350.44	0.01
K10118	msmF	raffinose/stachyose/melibiose transport system permease protein	ko02010 ABC transporters	M00196 Multiple sugar transport system	834.29	1391.00	0.01
K10563	mutM, fpg	formamidopyrimidine-DNA glycosylase [EC:3.2.2.23 4.2.99.18]	ko03410 Base excision repair		1287.81	330.33	0.005
K03517	nadA	quinolinate synthase [EC:2.5.1.72]	ko00760 Nicotinate and nicotinamide metabolism	M00115 NAD biosynthesis, aspartate => NAD	844.29	1324.67	0.01
K01695	trpA	tryptophan synthase alpha chain [EC:4.2.1.20]	ko00260 Glycine, serine and threonine metabolism<br>ko00400 Phenylalanine, tyrosine and tryptophan biosynthesis<br>ko01230 Biosynthesis of amino acids	M00023 Tryptophan biosynthesis, chorismate => tryptophan	765.43	1414.56	0.0004
K01811	yicI	alpha-D-xyloside xylohydrolase [EC:3.2.1.177]			776.95	1297.67	0.01
K10117	msmE	raffinose/stachyose/melibiose transport system substrate-binding protein	ko02010 ABC transporters	M00196 Multiple sugar transport system	769.67	1274.00	0.02
K01817	trpF	phosphoribosylanthranilate isomerase [EC:5.3.1.24]	ko00400 Phenylalanine, tyrosine and tryptophan biosynthesis<br>ko01230 Biosynthesis of amino acids	M00023 Tryptophan biosynthesis, chorismate => tryptophan	740.00	1343.11	0.0004
K01791	wecB	UDP-N-acetylglucosamine 2-epimerase (non-hydrolysing) [EC:5.1.3.14]	ko00520 Amino sugar and nucleotide sugar metabolism	M00362 Nucleotide sugar biosynthesis, prokaryotes	1094.10	488.00	0.005
K01657	trpE	anthranilate synthase component I [EC:4.1.3.27]	ko00400 Phenylalanine, tyrosine and tryptophan biosynthesis<br>ko01230 Biosynthesis of amino acids	M00023 Tryptophan biosynthesis, chorismate => tryptophan	733.05	1309.89	0.002
K00766	trpD	anthranilate phosphoribosyltransferase [EC:2.4.2.18]	ko00400 Phenylalanine, tyrosine and tryptophan biosynthesis<br>ko01230 Biosynthesis of amino acids	M00023 Tryptophan biosynthesis, chorismate => tryptophan	711.67	1332.67	0.0004
K07010		putative glutamine amidotransferase			1059.00	508.33	0.03
K01609	trpC	indole-3-glycerol phosphate synthase [EC:4.1.1.48]	ko00400 Phenylalanine, tyrosine and tryptophan biosynthesis<br>ko01230 Biosynthesis of amino acids	M00023 Tryptophan biosynthesis, chorismate => tryptophan	733.57	1243.56	0.002
K08316	rsmD	16S rRNA (guanine966-N2)-methyltransferase [EC:2.1.1.171]			706.48	1193.67	0.003
K08301	rng, cafA	ribonuclease G [EC:3.1.26.-]			720.14	1102.11	0.03

K07012	cas3	CRISPR-associated endonuclease/helicase Cas3 [EC:3.1.-.- 3.6.4.-]			644.81	1085.22	0.01
K03149	thiG	thiazole synthase [EC:2.8.1.10]	ko00730 Thiamine metabolism		588.81	1145.00	0.0004
K09963		hypothetical protein			1004.86	106.78	0.008
K01918	panC	pantoate--beta-alanine ligase [EC:6.3.2.1]	ko00410 beta-Alanine metabolism<br>ko00770 Pantothenate and CoA biosynthesis	M00119 Pantothenate biosynthesis, valine/L-aspartate => pantothenate	578.14	995.00	0.03
K03453	TC.BASS	bile acid:Na+ symporter, BASS family			530.81	1101.89	0.0004
K00857	tdk, TK	thymidine kinase [EC:2.7.1.21]	ko00240 Pyrimidine metabolism<br>ko00983 Drug metabolism - other enzymes		867.00	225.22	0.02
K01579	panD	aspartate 1-decarboxylase [EC:4.1.1.11]	ko00410 beta-Alanine metabolism<br>ko00770 Pantothenate and CoA biosynthesis	M00119 Pantothenate biosynthesis, valine/L-aspartate => pantothenate	512.14	910.89	0.008
K05847	opuA	osmoprotectant transport system ATP-binding protein	ko02010 ABC transporters	M00209 Osmoprotectant transport system	760.05	171.33	0.01
K01417	E3.4.24.-	putative zinc metalloprotease [EC:3.4.24.-]			689.33	200.67	0.04
K06310	yndD	spore germination protein			369.43	713.78	0.04
K06998	phzF	trans-2,3-dihydro-3-hydroxyanthranilate isomerase [EC:5.3.3.17]			633.43	96.11	0.04
K01118	acpD, azoR	FMN-dependent NADH-azoreductase [EC:1.7.-.-]			528.33	133.89	0.01
K02825	pyrR	pyrimidine operon attenuation protein / uracil phosphoribosyltransferase [EC:2.4.2.9]	ko00240 Pyrimidine metabolism		441.43	126.22	0.03
K00283	gcvPB	glycine dehydrogenase subunit 2 [EC:1.4.4.2]	ko00260 Glycine, serine and threonine metabolism<br>ko01200 Carbon metabolism		269.90	64.44	0.04
K09697	natA	sodium transport system ATP-binding protein	ko02010 ABC transporters<br>ko02020 Two-component system	M00253 Sodium transport system	264.38	71.89	0.02
K05970	E3.1.1.53, SIAE	sialate O-acetylesterase [EC:3.1.1.53]			90.29	410.56	0.02
K08296	sixA	phosphohistidine phosphatase [EC:3.1.3.-]			53.90	191.00	0.008
K00990	glnD	[protein-PII] uridylyltransferase [EC:2.7.7.59]	ko02020 Two-component system		45.38	179.56	0.008

Where available, information on KEGG pathways and/or modules is provided.
